# Temporal dynamics of the Rubber Hand Illusion

**DOI:** 10.1038/s41598-023-33747-2

**Published:** 2023-05-09

**Authors:** Gianluca Finotti, Sara Garofalo, Marcello Costantini, Dennis R. Proffitt

**Affiliations:** 1 Department of Psychology, University of Virginia, Charlottesville, Virginia USA; 2 Present Address: Center for Studies and Research in Cognitive Neuroscience, Department of Psychology, University of Bologna, Via Rasi e Spinelli 176, 47521 Cesena, FC Italy; 3 Department of Psychological, Health and Territorial Sciences, University G. d’Annunzio, Chieti-Pescara, Italy

**Keywords:** Human behaviour, Perception, Consciousness

## Abstract

It is widely accepted that the representation of the body is not fixed and immutable, but rather flexible and constantly updated based on a continuous stream of multisensory information. This mechanism can be very useful to adapt to several situations, but it would not be adaptive if the body representation was too malleable or if it wasn’t capable of restoring its integrity after a transient modification. Here we used the Rubber Hand Illusion (RHI) to investigate how quickly the body representation can be modified. Previous studies have investigated the timing of the onset and offset of the illusion, however, they did not assess a fine temporal resolution. Here, we used a potentiometer to record a moment-by-moment rating of the feeling of owning the RH for two minutes during the visuo-tactile stimulation and two minutes following the stimulation. Our results suggest that the feeling of Ownership is already established during the first 19 s of stimulation then it continues to grow, but at a much slower pace. The feeling of Ownership disappears within 66 s from the end of the stimulation. This work sheds new light on the temporal dynamics of the RHI and the malleability of the body self-consciousness.

## Introduction

Our body has a central role in a wide range of cognitive processes, shaping our self-awareness and our experience of the external world^1–3^. It is not surprising that there is a great interest in understanding how the brain generates a coherent representation of the body.

Since its introduction in 1998^4^, the Rubber Hand Illusion (RHI) has become the most popular method to experimentally test and manipulate the body representation. In a typical experimental design, a rubber hand is placed in front of the participant, close to their hand and facing the same direction. The experimenter uses two paintbrushes to synchronously stroke the rubber hand (in plain sight) and the participant’s hand (hidden from view). After a few seconds of stimulation, most participants experience that the touch they feel comes from the rubber hand^5,6^. Recently, this phenomenon has been explained within the theoretical framework of predicting coding^7,8^. According to this theory, the brain is constantly engaged in making predictions about the sensorial consequences evoked by events in the environment. These predictions are based on internal models and are constantly updated to minimize the level of surprise (or free-energy) across sensory systems^8,9^. A mismatch between a prediction about a sensory event and the sensory feedback elicited by the event generates a prediction error. When applied to the representation of the own body, the free-energy principles suggest that the mental representation of the self is also constructed in a probabilistic fashion, that is, what is more likely to be me is what I have learned to see in the mirror and what is associated with congruent, low-level sensory information, such as proprioceptive, interoceptive and tactile^7^. In the RHI, participants see the touch approaching the RH and have no reason to expect that this would evoke a sensation of touch on their hand because this is occluded from view. If the morphological properties of the RH are congruent with the stored representation of my own body and the feeling of touch occurs simultaneously with the seen touch, a prediction error arises in the somatosensory system which will be explained by top-down modulations from multisensory brain areas^10,11^ and by updating the representation of the own body^7^. In other words, the high-level probability that an external object belongs to one’s body increases and the probability that one’s hand is mine decreases. The RHI shows that the body representation is not fixed and immutable, but flexible and constantly updated according to the incoming sensory inputs^12^. However, the temporal dynamics of the RHI (how much time is needed for the illusion to grow to a maximum rating and to restore the integrity of the representation of the own body) are still poorly understood. The current experiment was designed to bridge this gap by assessing the temporal dynamics of the illusion—onset, growth, and decay—with a high temporal resolution.

Increasing our understanding of the temporal dynamics of the RHI is important for several reasons. As we said, one of the main findings achieved using the RHI is that the body representation is flexible and constantly updated. This can be useful in several situations, such as facilitating the use of a handheld tool^13,14^. However, this mechanism would not be adaptive if the body representation was too malleable or if it wasn’t capable of restoring its integrity after a transient modification. This notion is supported by previous research showing that the RHI is altered in some clinical populations. For instance, schizophrenic patients have a faster onset and stronger illusion compared to healthy controls^15,16^. Conversely, children with autism spectrum disorders have a delayed susceptibility to the RHI^17^. Thus, understanding the temporal dynamics of the RHI in healthy participants would help us understand how quickly the body representation can be modified and restored. In the future, this could potentially help us understand conditions in which the body representation is either too flexible or too immutable.

A few studies tried to clarify how long it takes for the illusion to emerge (e.g.,^18^) and how it changes over time (e.g.,^19^). However, these works lacked a fine-grained temporal resolution. For instance, Ehrsson and colleagues^20^ asked participants to press a keypad with their left foot when they started feeling that the rubber hand was their own and found that, on average, the illusion started after 14.3 ± 9.1 s. In another work, Kalckert and Ehrsson^18^ asked participants to verbally report the time point at which they felt that “the rubber hand was my hand” and found that the average onset time was below 30 s. Using a procedure similar to the one employed by Ehrsson^20^, Lloyd^21^ argued that the RHI can be reliably elicited in most participants (approximately eight out of ten) in less than 15 s. Perepelkina and colleagues^19^ investigated the onset and fading of a real and virtual RHI. To this aim, they adopted five stimulation periods (15, 30, 60, 120, and 240 s each) and measured proprioceptive drift and feeling of ownership at the end of each period. To investigate the fading of the illusion, after each stimulation phase there was an equally long period without tactile stimulation, at the end of which, the experimenter recorded again proprioceptive drift and feeling of ownership. However, their conclusion only showed that the RHI increases during the stimulation period and decreases over time during the post-stimulation period.

Notably, recent work by Abdulkarim and colleagues^22^ addressed the question of how long the illusion is maintained after the synchronous visuo-tactile stimulation stops. In two experiments, the authors first delivered the visuo-tactile stimulation for 60 s, then they measured the proprioceptive drift and the conscious feeling of illusion after the end of the visuo-tactile stimulation. The results from this study are very interesting as the authors found that the illusion persists for tens of seconds after the illusion stops (up to 300 s for the ownership ratings and 40 s for the proprioceptive drifts). Also, the authors show a similar pattern of decay for the conscious feeling of ownership and the proprioceptive drift. However, also in this work, the experience of ownership was not measured on a continuous scale but based on 5 different time intervals (i.e., 0 s, 20 s, 40 s, 60 s, 120 s, 300 s after the end of the visuo-tactile stimulation). Even though this study provides an idea of how the illusion changes over time, having only 6-time points over 300 s is a limitation when trying to gain insight into the temporal dynamics of the illusion. Summarizing, these experiments give us some important insights as to when participants start reporting illusory feelings of ownership and how these feelings change over time. Yet, mostly they focus on a single point in time in which participants report the beginning of the illusion and provide little or no insight into the online dynamics of the illusion. Moreover, the precise point in time in which the illusion disappears in the post-stimulation phase is still unknown.

Building on these prior studies, we first asked how the feeling of ownership changes over time during the visuo-tactile stimulation. Secondly, we asked how long it takes for the feeling of ownership to disappear once the experimenter stops delivering the visuo-tactile stimulation. Crucially, to gain an online measure of the illusion, we asked participants to continuously rate their feeling of ownership of the rubber hand during the entire duration of each phase by using a potentiometer (from now on, the ratings provided with the potentiometer will be referred to as “Ownership potentiometer ratings”). Based on past evidence, we predicted that the feeling of ownership would rapidly increase within the first 30 s of synchronous stimulation and then continue to increase at a slower pace until the end of the stimulation. For the post-stimulation phase, we predicted that the proprioceptive information coming from the real hand would quickly restore the body's integrity of the representation of the own limb, thus gradually reducing the feeling of ownership of the fake one.

## Results

### Proprioceptive drift and questionnaires

The results of the analyses on the proprioceptive drifts and questionnaires are reported in the supplementary materials. The analysis of the proprioceptive drift showed that participants had higher proprioceptive drifts in the rubber hand synchronous condition than in all other experimental conditions. Similarly, the analysis of the questionnaires showed higher scorings on the Embodiment and Ownership components of the illusion in the rubber hand synchronous condition. However, for the Ownership component, the 95% confidence interval touched zero ([0, 1.95]) and the CI was larger, indicating lower precision. These results indicate that the Ownership ratings tend to be higher in the RH synchronous condition, but some caution is required when interpreting the magnitude of this effect. This has potentially important implications given that this work focuses on the feeling of Ownership of the RH. For more on this, see also the Discussion section.

Overall, these results show that, at a group level, the RHI was successfully elicited as assessed both by implicit measures (proprioceptive drift) and explicit measures (RH questionnaire, especially Embodiment and Ownership). For more details, see the supplementary materials.

### Ownership potentiometer ratings

**Figure 1:**
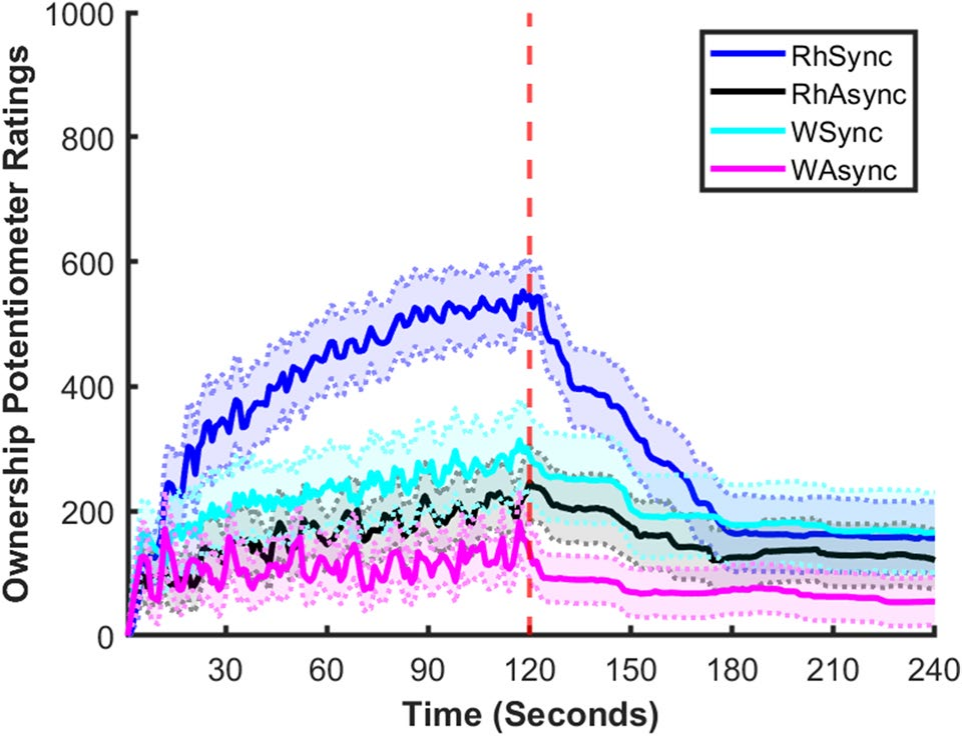
Shows the average ownership potentiometer ratings for the entire duration of the experiment in the four different conditions. The shaded bars show the standard error for each condition; the red dotted line shows the end of the visuo-tactile stimulation (120 s).

Figure 1 shows the Ownership Potentiometer Ratings averaged across all participants for the entire duration of the experiment. The range of possible answers went from 0 (no feeling that the viewed object is my hand at all) to 1000 (strong feeling that the viewed object is my hand). A visual inspection of this plot suggests that, despite some variability, on average participants reported stronger feelings of ownership during the rubber hand synchronous than in all other experimental conditions. This figure also shows that in this condition participants’ ratings rapidly increased during the first 30 s of stimulation and continued increasing until the stimulation was interrupted. At this point, Ownership Potentiometer Ratings start rapidly decreasing until there seems to be no difference between conditions.

It is important to notice that the observed magnitude of the Ownership Potentiometer Ratings is in line with previous studies. For instance, in a recent study Reader and colleagues^23^ examined the relationship between referral of touch and feeling of ownership in the RHI. To this aim, they re-analyzed three freely available datasets to better understand the relationship between referral of touch and feeling of ownership in the RHI. In particular, they report the ownership ratings in the RH Synchronous via boxplots from three studies^24–26^. In ^24^ and ^25^ the ownership was measured on a Likert scale from − 3 to + 3, and the median rating was 1 (see Fig. 1 in^23^), which corresponds to 57.14% of the scale, whereas in^26^, ownership was measured on a scale from 0 to 10 and the median rating was 5, which corresponds to 50% of the scale. This is in line with our study, in which the median Ownership Potentiometer Ratings between 90 and 120 s after removing outlier values was 553.13, corresponding to 55.31% of the scale (without excluding outlier values, the median was 548.33, that is 54.83% of the scale).

#### Stimulation phase

The following analyses aimed at investigating whether the Ownership Potentiometer Ratings in the RH synchronous differed compared to all other experimental conditions during and after the stimulation phase. First, we looked at the ratings during the visuo-tactile stimulation phase.

**Figure 2:**
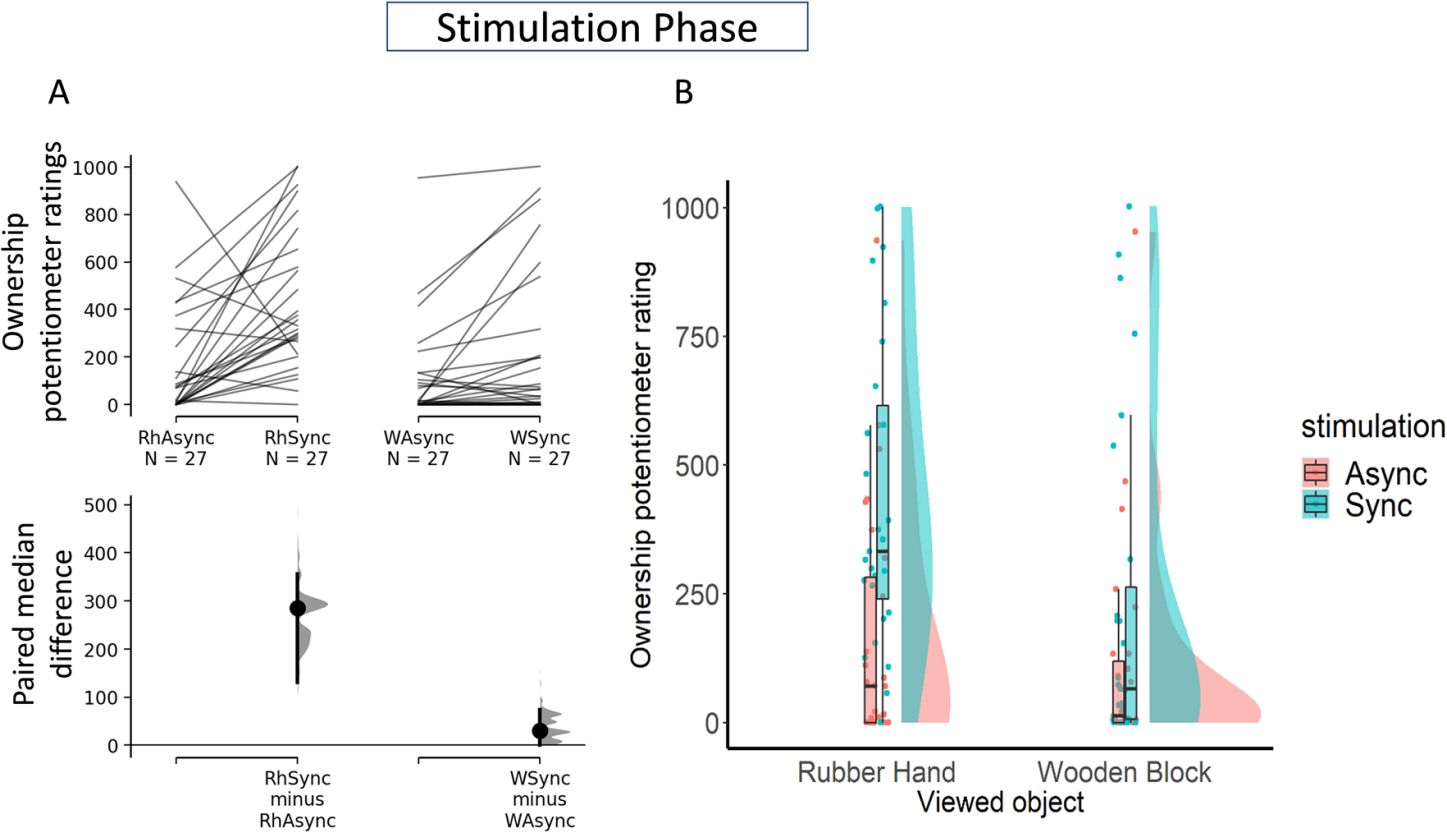
Panel (**A**) the upper axes show the raw data, that is, the Ownership Potentiometer Ratings averaged across 120 s of visuo-tactile stimulation; each paired set of observations is connected by a line. On the lower axes, the paired median difference between RH Synchronous and RH Asynchronous, and between Wood Synchronous and Wood Asynchronous is plotted as a bootstrap sampling distribution. Median differences are depicted as dots; 95% confidence intervals are indicated by the ends of the vertical error bars. Panel (**B**) A Raincloud plot shows the data distribution, the central tendency by boxplots, and the jittered raw data for the different experimental conditions for the 120 s of visuo-tactile stimulation.

With the rubber hand, there was a strong difference (M_diff_ = 285; 95% CI [126, 355]) between synchronous (Me = 332, IQR = 376) and asynchronous (Me = 70.07, IQR = 282) stimulation, indicating that the illusion was successfully induced in this condition (see Fig. 2A).

With the wooden hand, there was a weak difference (Me_diff_ = 30.3; 95% CI [0, 74.2]) between synchronous (Me = 65.1, IQR = 256) and asynchronous (Me = 12.7, IQR = 119) stimulation, indicating an absence of illusion in this condition (see Fig. 2A).

Overall, these results show that the Ownership Potentiometer Ratings during the visuo-tactile stimulation were higher in the RH synchronous as compared to all other experimental conditions, as can be seen with a raincloud plot (see Fig. 2B).

#### Post-stimulation phase

Here, we looked at the difference in Ownership Potentiometer Ratings during the post visuo-tactile stimulation phase.

**Figure 3:**
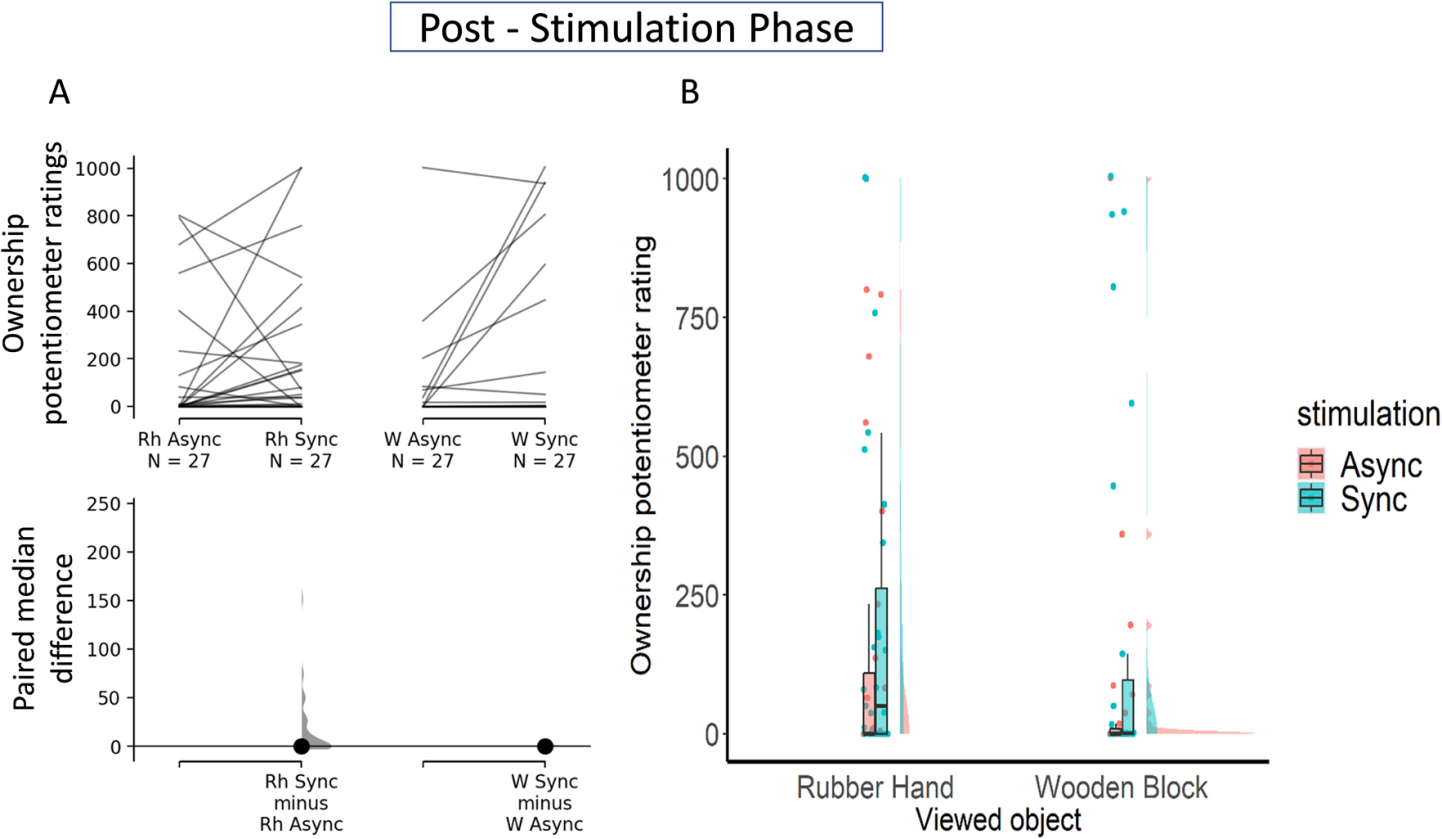
Panel (**A**) the upper axes show the raw data, that is, the Ownership Potentiometer Ratings averaged across 120 s post visuo-tactile stimulation; each paired set of observations is connected by a line. The lower axes show the paired median difference between RH Synchronous and RH Asynchronous, and between Wood Synchronous and Wood Asynchronous. Panel (**B**) A Raincloud plot shows the data distribution, the central tendency by boxplots, and the jittered raw data for the different experimental conditions for the 120 s post-visuo-tactile stimulation.

With the rubber hand, there was no difference (Me_diff_ = 0.0; 95% CI [− 2.77, − 2.77]) between synchronous (Me = 50.4, IQR = 263) and asynchronous (Me = 0, IQR = 107) stimulation, indicating an absence of illusion in this condition (see Fig. 3A).

Similarly, with the wooden block, there was no difference (Me_diff_ = 0; 95% CI [0, 0]) between synchronous (Me = 0, IQR = 97.5) and asynchronous (Me = 0, IQR = 9.36) stimulation, indicating an absence of illusion (see Fig. 3A).

Overall, in the post-stimulation phase, there was no difference between the RH synchronous and all other experimental conditions, as can be seen with a raincloud plot (see Fig. 3B).

#### Progression over time during stimulation

Here, we looked at the progression over time of the Ownership Potentiometer Ratings during the stimulation phase with the rubber hand.

**Figure 4:**
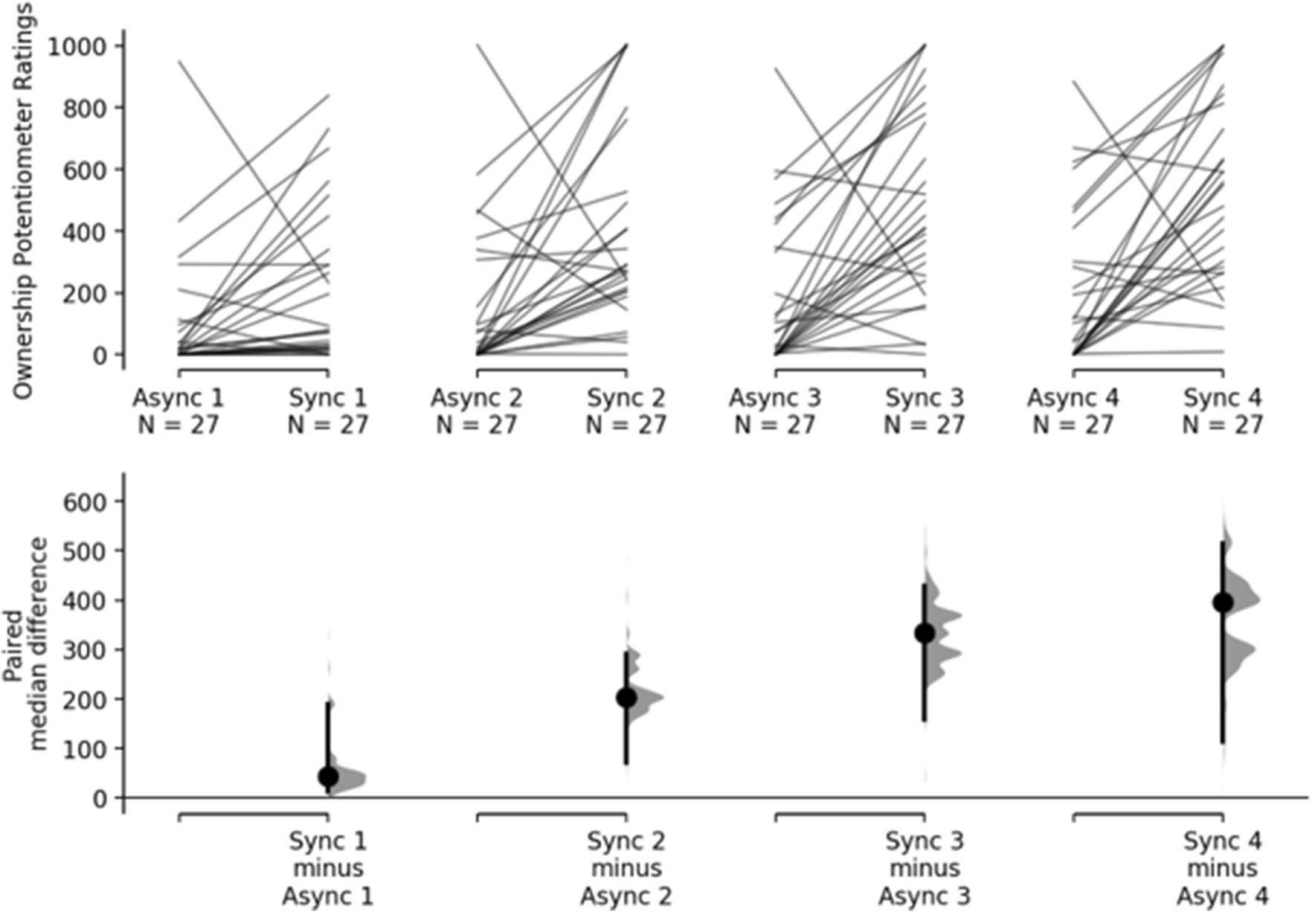
The upper axes show the raw data, that is, the Ownership Potentiometer Ratings for the 120 s of visuo-tactile stimulation averaged across 4, 30 s blocks. Each paired set of observations is connected by a line. The lower axes show the paired median difference between RH Synchronous and RH Asynchronous for each time-block.

Results of the multiple paired estimation statistics showed that the difference between the synchronous and asynchronous conditions was weak at time 1 (Me_diff_ = 44.3; 95% CI [12.5, 180]), moderate at time 2 (Me_diff =_ 203; 95% CI [71.6, 291]), strong both at time 3 (Me_diff_ = 333; 95% CI [159, 429]) and at time 4 (Me_diff_ = 396; 95% CI [115, 514]) (see Table 1 and Fig. 4).

**Table 1:** Shows the median and interquartile range for the Rubber Hand Synchronous (second column) and the Rubber Hand Asynchronous (third column).

Time	RH Sync median, IQR	RH Async median, IQR
1: 0–30 s	78.8, 294	16.6, 83.2
2: 31–60 s	271, 304	24.1, 231
3: 61–90 s	412, 517	71.6, 340
4: 91–120 s	548, 497	102, 356

Each row refers to a different time block of visuo-tactile stimulation (1 to 4 with each time block lasting 30 s).

Summarizing, during the visuo-tactile stimulation, Ownership Potentiometer Ratings already differ at time 1, although this difference is weak. Cumming estimation plots also show that the difference between the two conditions increases over time becomes strong at time 3 and is maximum at time 4, that is, during the last 30 s of stimulation.

#### Progression over time post-stimulation

Here, we looked at the progression over time of the Ownership Potentiometer Ratings during the post-stimulation phase with the rubber hand.

**Figure 5:**
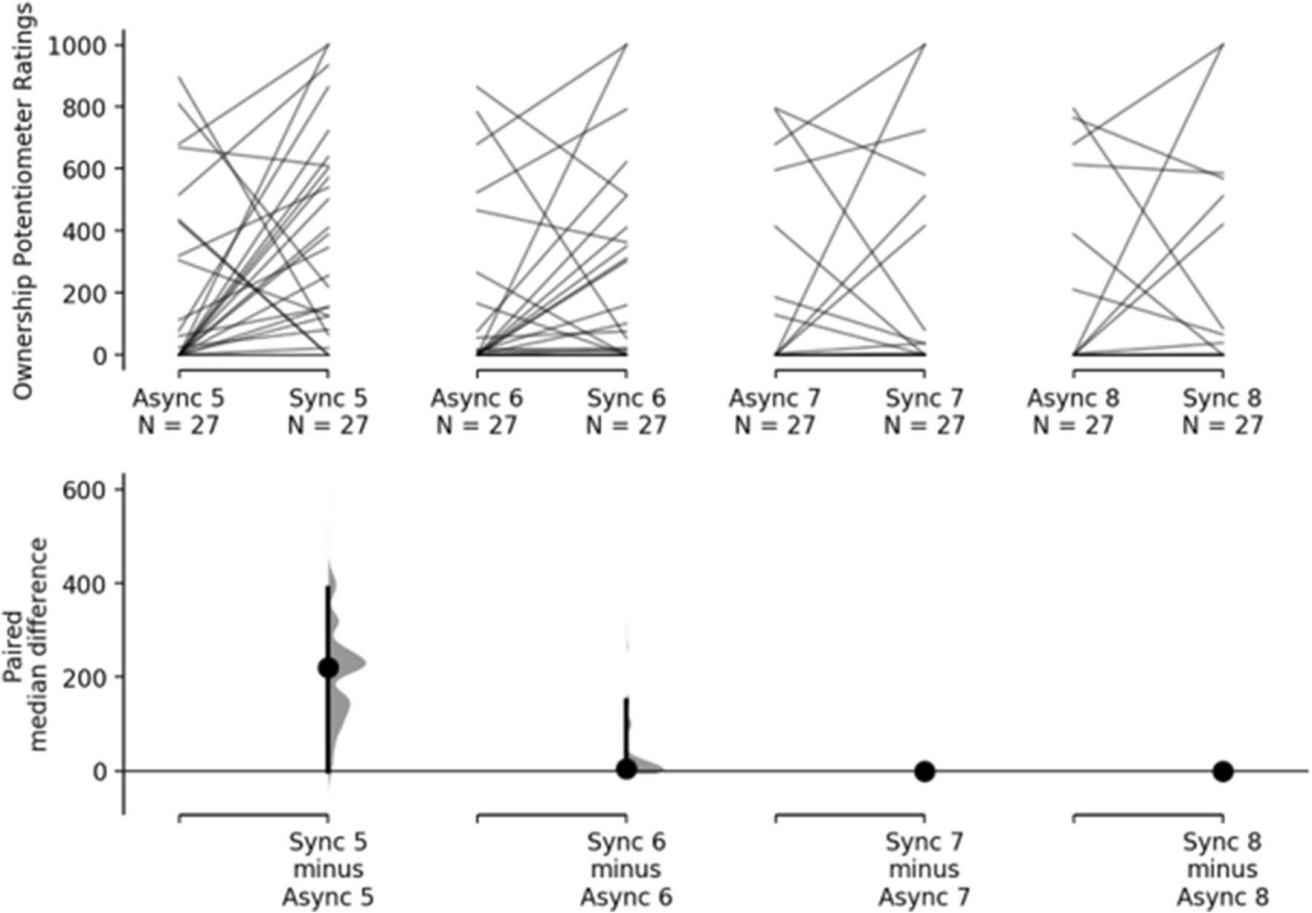
The upper axes show the raw data, that is, the ownership potentiometer ratings for the 120 s post-visuo-tactile stimulation averaged across 4, 30 s blocks. Each paired set of observations is connected by a line. The lower axes show the paired median difference between RH Synchronous and RH Asynchronous for each time-block.

Results of the multiple paired estimation statistics showed that the difference between the synchronous and asynchronous conditions was moderate at time 1 (Me_diff_ = 220; 95% CI [0, 389]), weak at time 2 (Me_diff_ = 4.93; 95% CI [0, 149]), and there was no difference both at time 3 (Me_diff_ = 0; 95% CI [0, 0]) and at time 4 (Me_diff_ = 0; 95% CI [0, 0]) (see Table 2 and Fig. 5).

**Table 2:** Shows the median and interquartile range for the Rubber Hand Synchronous (second column) and the Rubber Hand Asynchronous (third column).

Time	Rh Sync median, IQR	Rh Async median, IQR
1: 121–150	345, 503	0, 374
2: 151–180 s	75.6, 387	0, 121
3: 181–210 s	0, 58.8	0, 65.9
4: 211–240 s	0, 75.6	0, 1.52

Each row refers to a different time block of visuo-tactile stimulation (1 to 4 with each time-block lasting 30 s).

Summarizing, results show that, once the visuo-tactile stimulation is interrupted, Ownership Potentiometer Ratings are still higher for the RH Synchronous as compared to the RH Asynchronous during the first 30 s, the difference becomes smaller between 30 and 60 s and disappears after 60 s.

#### Changepoint analysis during stimulation

Here, we report the results of the changepoint analysis on the Ownership Effect (which was calculated by subtracting the average Ownership Potentiometer Ratings in the RH asynchronous from the RH synchronous condition). The Changepoint analysis was set to find two changepoints for each phase, stimulation and post-stimulation.

**Figure 6:**
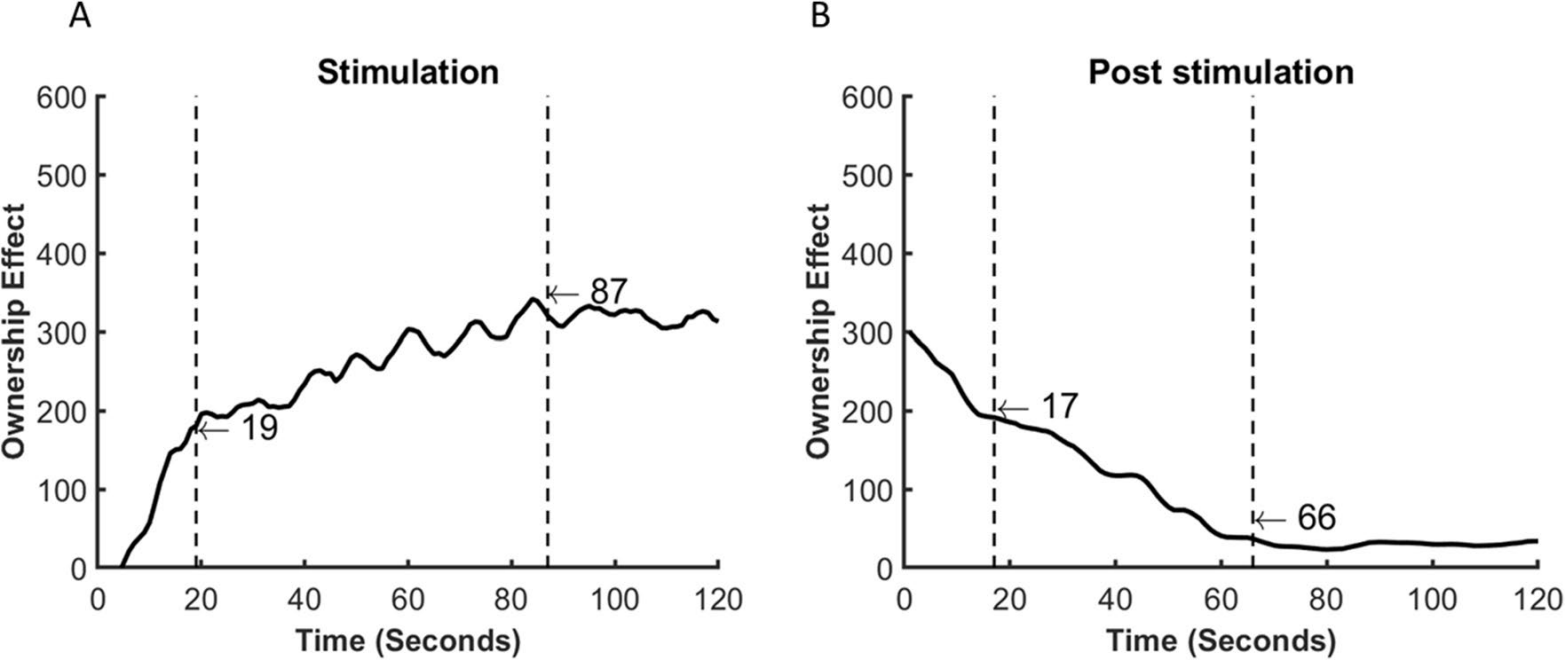
Panel (**A**) shows the average Ownership Effect during the 120 s of visuo-tactile stimulation in the rubber hand synchronous condition. In particular, the plot shows the two points where the Ownership Effect changes most abruptly. The vertical dashed lines separate the regions between changepoints. Panel (**B**) shows the same information but refers to the 120 s post-visuo-tactile stimulation.

**Figure 7:**
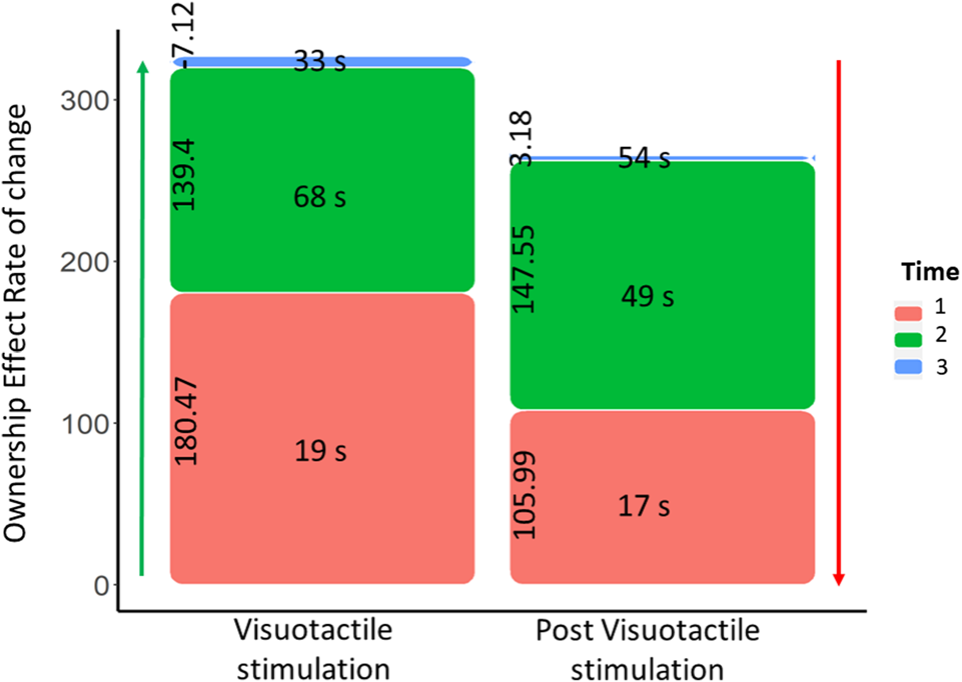
Shows the rate of change in the Ownership Effect during and post-visuo-tactile stimulation. The timepoint analysis divided each phase into 3 time-periods of different lengths. The colour of the boxes indicates the order of the time-periods. The horizontal number inside each box shows the number of seconds between changepoints, whereas the vertical number shows the amount of rating change during that time. The vertical arrows next to the barplots show whether the ratings were increasing (green arrow) or decreasing (red arrow). The Y axis shows the total amount of change in ownership potentiometer ratings for the 3 timeblocks taken together.

**Figure 8:**
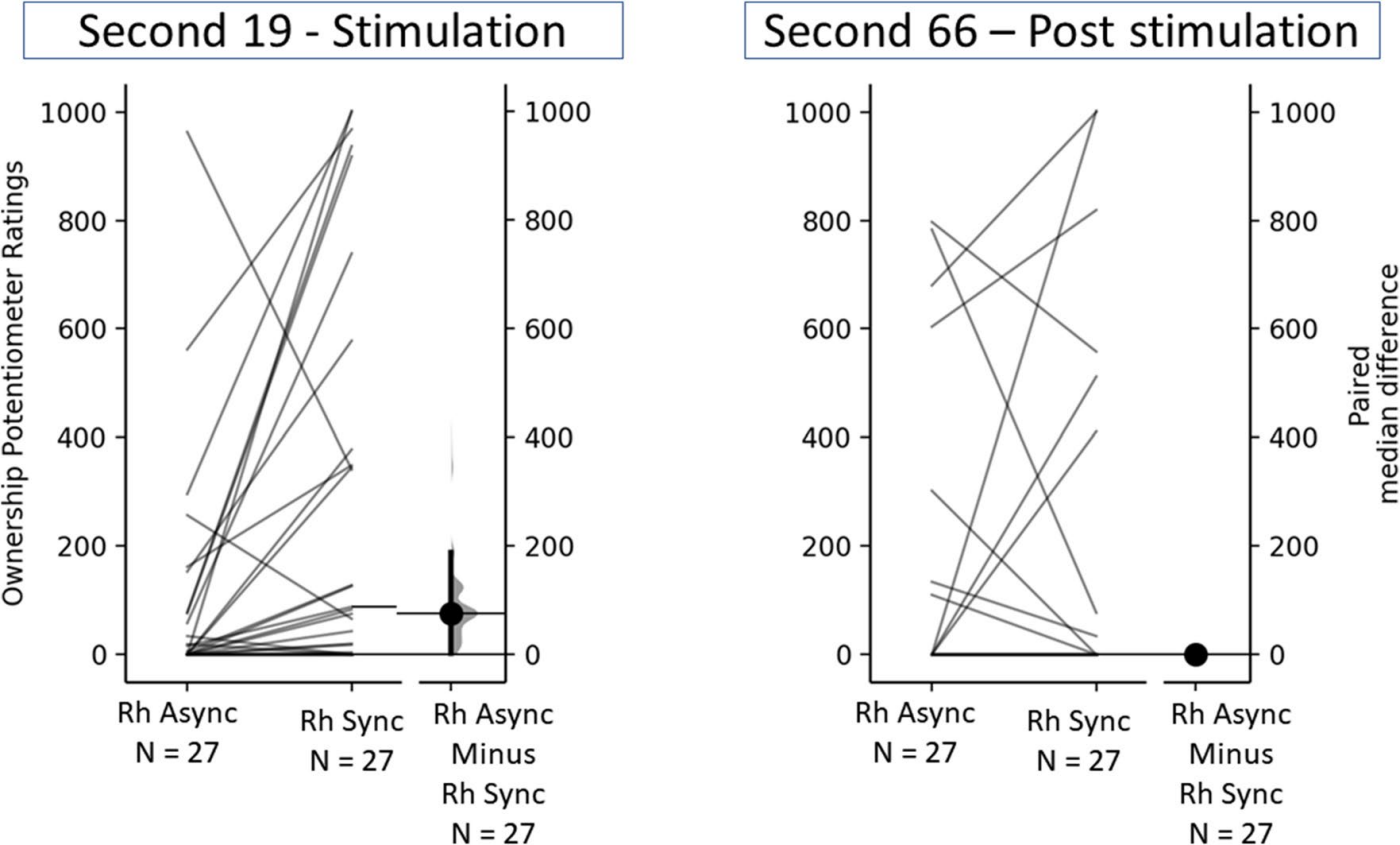
The upper axes show the raw data, that is, the Ownership Potentiometer Ratings at 19 s from the beginning of the visuo-tactile stimulation (Left Panel) and 66 s from the end of the visuo-tactile stimulation (Right Panel); each paired set of observations is connected by a line. The lower axes show the paired median difference between RH Synchronous and RH Asynchronous.

During the stimulation phase, the two changepoints were found at 19 and 87 s (see Fig. 6A). This shows that, on average, participants start experiencing a feeling of ownership of the RH soon after the beginning of the visuo-tactile stimulation, as can be seen by the steep increase of the Ownership Effect from time 0 to the first changepoint at 19 s. In particular, during this first period (0 to 19 s), the average Ownership Effect goes from 0 to 180.47 points. After 19 s, the Ownership Effect continues to increase but at a slower pace until the second changepoint at 87 s, where it reaches on average 319.87 points. After 87 s, the Ownership Effect becomes stable, fluctuating between 300 and 320 points and reaching an average of 312.75 points at 120 s. In other words, in our data, in the first 19 s, the Ownership Effect increased by 180.47 points, in the following 68 s they increase by 139.4 points and in the last 33 s, it decreases by 7.12 points (see Fig. 6A). This seems to indicate that, while continuing the stimulation increases the feeling of ownership, most of this feeling is already established during the first 19 s of stimulation (see Fig. 7, left bar). The comparison of the Ownership Potentiometer Ratings between the RH Synchronous (ME = 88, IQR = 467) and the RH Asynchronous (ME = 1, IQR = 77) at 19 s seems to at least partially support this interpretation, showing evidence of a weak difference between these conditions at this timepoint (Me_diff_ = 75; 95% CI: [1, 18] see Fig. 8, left panel).

#### Changepoint analysis post-stimulation

The changepoints analysis of the post-stimulation period found the two changepoints at 17 and 66 s (see Fig. 6B). This shows a rapid decline in the Ownership Effect between the first and the second sector, up to the 66th second. From this point on, the Ownership Effect seems to almost completely disappear with the effect fluctuating close to 0.

In particular, in the first 17 s post-stimulation, the Ownership Effect goes from 299.34 to 191.20 points; it reaches 36.92 at 66 s. From this point, the effect seems to stabilize close to zero (between 25 and 35 points). In other words, the Ownership Effect decreases by 108.1 points in the first 17 s, by 154.28 points in the next 49 s, and effectively becomes stable after that point (see Fig. 7, right bar). This interpretation is further supported by the comparison of the Ownership Potentiometer Ratings between the RH Synchronous (ME = 0, IQR = 55.5) and the RH Asynchronous (ME = 0, IQR = 56.5) at 66 s post-stimulation, showing no evidence of a difference between these conditions (Me_diff_ = 0 [95%CI 0, 0]; see Fig. 8, right panel).

Summarizing, results from these analyses suggest that the feeling of ownership of the rubber hand rapidly increases after the beginning of the visuo-tactile stimulation and keeps increasing for around 68 more seconds, at which point it becomes stable until the stimulation is interrupted. After the interruption, the feeling of ownership rapidly decreases in the first 17 s and reaches almost zero after 66 s. Moreover, these analyses, while not conclusive, provide evidence that the feeling of ownership is already established during the first 19 s of stimulation and disappears after the 66 s post-stimulation.

#### Individual changepoints

**Figure 9:**
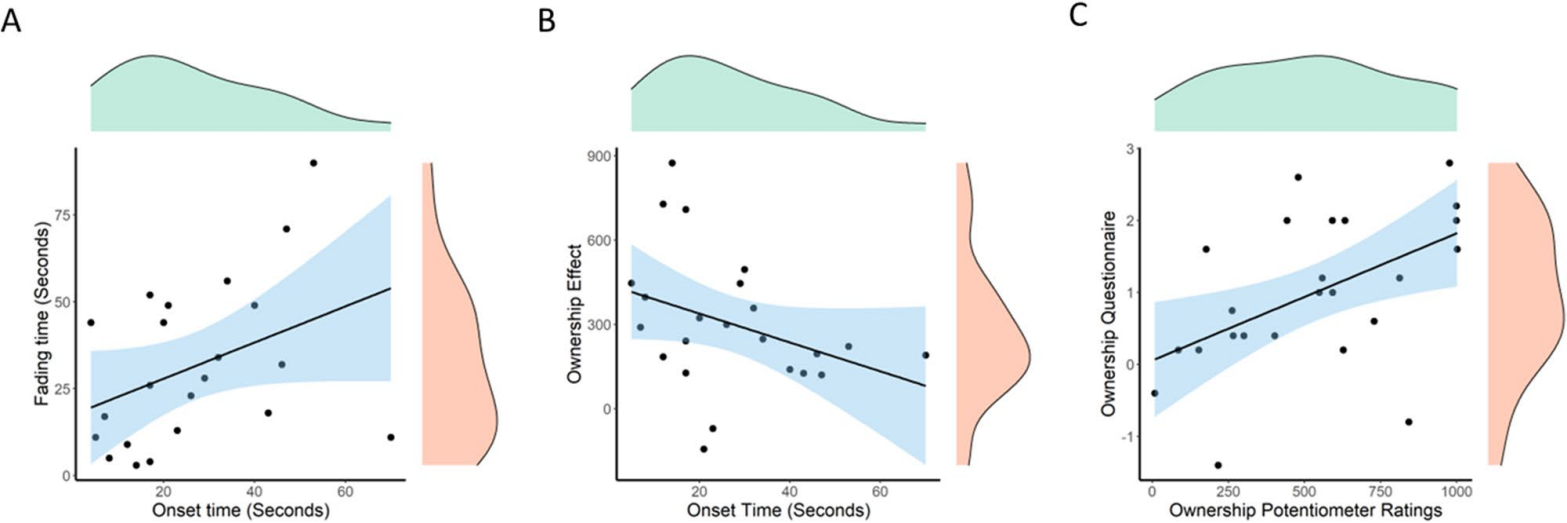
Shows the relationship between the first of the onset changepoints (during visuo-tactile stimulation) and (**A**) the first of the fading changepoints (post-stimulation); (**B**) the Ownership effect averaged during the two minutes of visuo-tactile stimulation in the rubber hand synchronous condition. Notice that the changepoints were calculated separately for each participant and the Ownership Effect was calculated by subtracting the RH Asynchronous from the RH Synchronous Ownership Potentiometer Ratings. (**C**) shows the relationship between the Ownership Potentiometer Ratings between 90 and 120 s of visuo-tactile stimulation and the Ownership ratings measured with the RHI questionnaire in the RH Synchronous condition.

The following analyses focus on the changepoints calculated on the Ownership Effect for each participant. We refer to the first changepoints during the visuo-tactile stimulation period as the Onset Changepoints and the first changepoints during the post visuo-tactile stimulation period as the Fading Changepoints. In particular, we investigated the relationship between the Onset Changepoints and: (1) the Fading Changepoints (Fig. 9A); (2) the average Ownership Effect (Fig. 9B); (3) the Embodiment scores as measured via questionnaires; (4) the proprioceptive drifts. The results of the correlations are reported in Table 3.

**Table 3:** Shows the results of the correlations between the individual Onset Changepoints and (1) fading changepoints; (2) ownership effect; (3) ownership questionnaires and (4) proprioceptive drifts.

Second term of the correlation	N	Onset changepoints ME/IQR	Corr. second term ME/IQR	r_s_	BCa 95% CI	Uncorr. p	Corr. p
Fading changepoints	22	22/23.75	27/36.25	0.45	− 0.17, 0.76	0.032	0.192
Ownership effect	23	23/21.5	248.23/259.12	− 0.43	− 0.69, − 0.07	0.037	0.222
Ownership questionnaires	22	22/18.75	1.1/1.6	0.08	− 0.40, 0.48	0.7	4.2
Proprioceptive drifts	21	21/22	2/4	0.33	− 0.61, 0.19	0.33	1.98

For each correlation, we report the N = number of participants after removing outlier values; Onset Changepoints ME/IQR = Median and Interquartile Range for the Onset Changepoints; Corr. Second term ME/IQR = Median and Interquartile for the second term of the correlation (first column of the table); r_s_ = Spearman correlation coefficient; BCa 95% CI = Bias Corrected accelerated 95% Confidence Interval; Uncorr.p = uncorrected p-value; Corr.p = p-value after Bonferroni Correction for multiple comparisons.

### Relation to classical measures of illusion

#### Ownership questionnaire

The correlation (N = 22) performed between Ownership Potentiometer Ratings during the two minutes of visuo-tactile stimulation (ME = 332.10, IQR = 375.67) and the Ownership component of the illusion as measured with the questionnaires (ME = 0.75, IQR = 1.6) was not significant r_s_ = 0.29, BCa 95% CI [− 0.14, 0.61], p = 0.13 (p = 0.78 after Bonferroni correction for multiple comparisons). However, the correlation (N = 24) between the Ownership Potentiometer Ratings during the last 30 s of visuo-tactile stimulation (seconds 90 to 120; ME = 553.13, IQR = 485.43) and the Ownership component of the illusion as measured with the questionnaires was significant (ME = 1, IQR = 1.65) r_s_ = 0.51, BCa 95% CI [− 0.07, 0.81], p = 0.009, and showed a trend after Bonferroni correction for multiple comparisons, p = 0.054 (see Fig. 9C).

## Discussion

The present study investigated the temporal dynamics of the Rubber Hand Illusion (RHI). Our main goal was to investigate how the conscious experience of body ownership of the rubber hand changes after the visuo-tactile stimulation. With a novel technique based on the use of a potentiometer, for the first time, to the best of our knowledge, participants were able to continuously rate the strength of the illusion during and after the stimulation (what we refer to as “Ownership potentiometer ratings”).

First, we show that this novel technique was able to capture the classic effect of the RHI as shown by higher Ownership Potentiometer Ratings during the RH synchronous stimulation as compared to all other experimental conditions. The analyses on the stimulation period are compatible with the idea that, on average, the illusion is established in the first 30 s of stimulation and it increases over time until the stimulation is interrupted. This is in line with previous experiments, indicating that 30 s of stimulation are enough to induce the illusion in 70% of healthy subjects^5,21,27^. Our data go further in showing the progress of the illusory feeling of ownership with higher temporal resolution. The Ownership Effect (i.e., the difference in Ownership Potentiometer Ratings between the RH Synchronous and the RH Asynchronous) increased immediately after the beginning of the visuo-tactile stimulation. The fastest rise in the Ownership Effect happens in the first 19 s. In line with this, the total increase in the ratings during the following 101 s (132 points) is smaller than the increase during the first 19 s (180 points). This suggests that the illusory feeling of ownership keeps increasing, as expected, throughout the stimulation while the rate lowers over time and becomes stable, on average, after 87 s from the beginning of the stimulation.

The most novel finding pertained to the post-stimulation phase. Results showed that taken together, in the two minutes post-stimulation there is no difference in the Ownership Potentiometer Ratings between the Rubber Hand Synchronous and all other experimental conditions, potentially suggesting that, in absence of stimulation, the feeling of owning the RH disappears within two minutes. The progression analysis of the Ownership Potentiometer Ratings in the RH Synchronous shows that the feeling of ownership rapidly decreases after the visuo-tactile stimulation is interrupted, however, on average, it only fully disappears after 1 min without stimulation. The changepoint analysis conducted on the Ownership Effect post-stimulation shows that, again, the fastest decrease happens during the first 17 s after which the Ownership Effect continues to rapidly decrease and fluctuates close to 0 after 66 s without stimulation.

It is important to notice that, even though the Ownership Potentiometer Ratings were statistically higher in the RH Synchronous than in all other experimental conditions, the magnitude of the ratings was not very high, reaching, on average, approximately 50% of the scale. However, we believe that this is in line with previous studies. As we mentioned in the results section, previous studies investigating the Ownership of the RH via questionnaires found that, at a group level, the median Ownership ratings fell between 50 and 70% of the scale^24–26^. This is in line with our study, in which the median Ownership Potentiometer Ratings in the 90 s to 120 s interval, when the ratings were at the highest, reached 55% of the scale. It is also worth noting that the RHI questionnaire Ownership ratings observed in our study were also in line with previous studies (the median in the synchronous condition was 0.75, which corresponds to 67.85% of the scale). In our opinion, the observed magnitude of Ownership ratings provided both with the potentiometer and the RHI questionnaires should not be surprising given the characteristics of this illusion: the RHI is a particular type of illusion given that the feeling that the RH belongs to one’s body is never complete. This can be observed in the magnitude of the responses to the RHI questionnaires, as in the works just mentioned, and also in the fact that the proprioceptive drift is on average only about 15–30% of the total distance between the real hand and the RH^5^.

The results of the correlations also offer some suggestive insights. In particular, thecorrelation between the first onset changepoints and the first fading changepoints seems to indicate that participants who started having feelings of ownership more quickly might also be quicker in losing this feeling. If true, it could mean that these participants have a higher malleability of the body representation, that is, on one side they can incorporate an external object more quickly, and on the other side they also restore the integrity of the body representation more quickly. However, it should be noted that this correlation was conducted on a small number of participants (N = 22) and that it was not significant after correction for multiple comparisons. The same is true for the correlation between the first Onset Changepoint and the average Ownership Effect during the two minutes of stimulation. This correlation seems to suggest that those participants whose initial increase in the feeling of Ownership tended to last longer, also had an overall higher Ownership Effect. Again, this correlation was conducted on a small sample and the results do not resist a correction for multiple comparisons.

It is also interesting to notice that the Ownership Potentiometer Ratings averaged over the two minutes of RH Synchronous stimulation did not correlate with the Ownership ratings in the same condition as measured via the questionnaires. However, when considering only the average ratings during the last thirty seconds of stimulation, these two measures tended to correlate: participants who rated the Ownership feelings as higher in the questionnaire tended to also give higher Ownership ratings during the last 30 s of stimulation. It is reasonable to expect that, when answering the questionnaires, participants rate the illusion based on when it was at its peak. This relation between the Ownership Potentiometer Ratings and a well-established measure of the illusion such as the questionnaires further suggests that the use of a potentiometer is a viable method for measuring the feeling of ownership during the stimulation. However, once again, this correlation was conducted on a rather small sample (N = 24) and it was not significant after Bonferroni correction (p = 0.054).

In this regard, we believe that in this case, it is not appropriate to correct for multiple comparisons. These corrections are usually applied to reduce the probability of false positives (type I error). However, this approach has several limitations, most prominently, it increases other sources of error, like the chance of false negatives (type II error). It has been argued that correcting for multiple comparisons is more appropriate when carrying out exploratory correlations (not hypothesis-driven) than when carrying out hypothesis-driven correlations, as in our case (for more on this, see^28^). As this is a matter of debate, we have reported both corrected and uncorrected p-values.

Beyond showing the progression of the feeling of ownership through time, we believe that the present results also show the potential of this novel technique to further our understanding of the RHI and, in turn, of the malleability of the body representation. For instance, future experiments could use this method to investigate the temporal dynamics of the other components of the illusion. Longo and colleagues found that the second component in terms of variability explained is the feeling of losing one’s hand^29^, further confirmed by recent findings from Romano and colleagues^30^. One interesting possibility would be to investigate the temporal dynamics of this component of the illusion and whether it follows a similar pattern as the feeling of ownership. Even more interestingly, this technique could be employed to investigate how participants differ in their experience of the illusion. It is well known that there is a high interindividual variability in the susceptibility to the illusion: some participants (around 30% of the population^5^) seem to be immune to the illusion and, of those who experience it, some report very mild and others very strong and vivid illusory feelings, as can be seen also in the present data. Many studies have investigated the causes of this variability (e.g.^31–33^) however, these are still largely unknown. Similarly, it is still a matter of debate why some clinical populations tend to be more or less susceptible to the RHI as compared to healthy participants (e.g.^17,34–37^). Classic measures such as the RHI questionnaires, proprioceptive drift and physiologic measures such as skin conductance have proven extremely valuable as indices of the magnitude of the illusion. By recording the moment-by-moment development of the feeling of ownership, future experiments could draw an even more complex picture giving us access to the inner dynamics of this feeling as it develops through time.

This is particularly important given that, since its introduction, a wealth of studies has been published trying to clarify under which conditions the illusion is established, and it has been clear that the temporal parameters are some of the most important to successfully elicit the illusion. There are several temporal parameters, among the most important are: (1) the temporal discrepancy between the visual and the tactile stimulation, and (2) the duration of stimulation. We argue that a systematic investigation of these temporal parameters will be crucial to fully understand the RHI and the plasticity of the bodily self-consciousness and we suggest that drawing inspiration from the literature on Classical Pavlovian Conditioning would be extremely valuable to achieve this goal, given that the investigation of these two temporal parameters has also been central in the related literature.

As we explained in the introduction, current theories such as the free-energy principles suggest that the body representation is built in a probabilistic fashion, by integrating top-down processes, for instance, what I have learned to see in the mirror, and low-level processes, such as multisensory associations learned throughout development^7^. Recent studies have shown that experiences with learned associations between multisensory stimuli and their statistics are crucial in the processing of multisensory stimuli and whether they will be integrated (e.g.^38–40^). The RHI is an instance of a larger range of multisensory integration phenomena investigated in the literature on Classical Conditioning, a type of learned association between two stimuli. In its most simple form, in Classical Conditioning, there is a stimulus that can induce a measurable response from the first time it is presented (Unconditioned Stimulus, US) and a stimulus that, at first, doesn’t elicit a response (Conditioned Stimulus, CS). After repeated presentations of the CS followed by the US, the CS begins eliciting a conditioned (learned) response (for an overview of the history of classical conditioning see^41^).

The extensive literature on Classical Conditioning has focused on the temporal parameters involved in the establishment of this association, such as (1) the temporal discrepancy between the US and the CS, and (2) the number of pairings necessary for the acquisition. For instance, already Pavlov found that increasing the temporal distance between the US and the CS led to a reduced conditioned response^42^. Also, some associations build up gradually, such as the association between sound and taste in Pavlov’s experiments^41^, some in one trial, such as taste aversion (the learned association between a poisonous food and its taste^43,44^), and others never do, such as the association between a poisonous food and sound^45^. Given the importance of temporal dynamics in the literature on Classical Pavlovian Conditioning, we believe that research on the RHI would benefit from a similar research program. More broadly speaking, we propose that the RHI can be considered an expression of associative learning and, therefore, a theoretical perspective accounting for this link would be timely and valuable. Following this line of reasoning, in our current study, we look at the time course of associative learning between two stimuli (touch and sight). Therefore, we propose that a critical variable to understanding classically conditioned learning is to assess the time course of learning. For instance, similarly to what has been done in Classical Conditioning, future studies should elucidate what is the most effective variable for the buildup of the illusion: time or number of stimulations and the interplay between several factors, such as time (i.e., duration of the stimulation), number of visuo-tactile pairings, the temporal delay between pairings and the velocity of touch.

One way in which future experiments could clarify the relative role of these factors would be by using a fully automated setup. Previous experiments have already employed servo or stepper motors to deliver the visuo-tactile stimulation and have repeatedly demonstrated that this approach can successfully elicit the illusion (e.g.^6,12,46^). These automated methods are relatively inexpensive and easy to implement and allow the experimenter to accurately control virtually every aspect of the visuotactile stimulation, such as the overall duration of the stimulation, the duration of contact between the brush and the hands, the delay between touches and number of touches, to mention a few. In the future, it will be important to capitalize on these automated methods to clarify the role of time in the RHI and the interplay between its several components and how these determine the temporal dynamics of the illusion. In this regard, it is also interesting to notice that many studies have demonstrated that the RHI can be elicited with different types of stimulation other than visuo-tactile, such as finger movements of one’s hand and the RH (e.g.^47–49^), ultrasound^50^, and vibrotactile stimulation^51^. In the future, it will be interesting to investigate whether the RHI induced with these types of stimulation shares the same temporal dynamics as the RHI induced with more classical types of stimulation such as the one investigated in this work.

Understanding the temporal dynamics of the RHI is important because it could increase our understanding of how the representation of the own body can adaptively change moment-by-moment, for instance during tool use (e.g.^52,53^). Moreover, as we mentioned above, there is growing evidence that the malleability of the body representation is altered in conditions such as autism and schizophrenia (^15,16,17^). An investigation of the temporal dynamics of the RHI in healthy participants and clinical populations could help us understand whether and how the malleability of the body differs over time in clinical patients and whether, for instance, feelings of ownership last longer or less in these populations as compared to healthy subjects. This, in turn, could shed light on how the underlying processes, such as multisensory integration, are modified in patients.

In addition, there is also some interest in using the RHI as a tool in rehabilitation, in particular with amputees suffering from phantom sensations and chronic pain. In this respect, the RHI might facilitate the integration of an external prosthesis into the body image (e.g.^54^). Knowing the temporal dynamics of the RHI and how long it takes for the illusion to be established and to fade after the visuo-tactile stimulation would be important to investigate its potential application as a rehabilitation tool. For instance, knowing that, on average, most of the illusion is already established during the first 20 s of stimulation is important for paradigms that require short periods of stimulation.

Some important limitations should be taken into account when evaluating the present results. Some of these limitations have already been mentioned, most prominently: all the correlations were conducted on a rather small sample size and none of them was significant after correction for multiple comparisons. However, as we argued above, we believe that in this case it is not appropriate to apply such corrections. Another limitation is that our sample was imbalanced for gender, with 22 females and 5 males included in the final analyses. This may have affected the generalizability of our results. Future experiments should further test whether there are any differences in the progress of the illusion between males and females. Also, as we mentioned, for about ten seconds post-stimulation participants were engaged in the proprioceptive judgement and could not provide the Ownership Potentiometer Ratings, however, we have not measured the exact length of time each participant took. With this experiment, it is therefore not possible to know what participants were feeling in the moments immediately following the ending of the stimulation and whether this affected subsequent ratings. One more thing that should be noted is that, at a group level, the feeling of Ownership established in our participants was not very high as suggested by the magnitude of both the Ownership Potentiometer Ratings and the analysis of the Ownership component of the RHI questionnaires, even though the overall scorings for both these measures are in line with previous studies. Nonetheless, it is possible that if higher ratings of Ownership had been achieved, this could have in turn affected and possibly delayed the reduction in the Ownership Effect that we observed in the post-stimulation. Another potential confound regarding the use of the potentiometer is that the potentiometer started at zero for all participants. This assumes that participants have no immediate feelings of ownership driven by visual dominance over proprioception. A possible solution to this issue in future studies could be to use a digital slider. This would allow depicting the entire scale without depicting the position of the dial from the beginning.

Another important point concerns the choice of using the RH Asynchronous as the main control condition. This choice is in line with virtually all the RH illusion experiments present in literature, but it has been recently challenged in a series of works arguing that the RHI is affected by demand characteristics and that the asynchronous stimulation is a poor control condition^55–57^. Lush and colleagues found that both for indirect measures such as the proprioceptive drifts^56^ and direct measures such as the questionnaires^55^ participant’s expectations were higher in the Synchronous than the Asynchronous conditions. Moreover, the authors proposed that the RHI may be mainly caused by hypnotic suggestibility rather than by multisensory integration^58^. This has sparked a lively debate regarding the methodology and interpretation of the RHI, with other authors challenging this view (e.g.^59–61^). It is not the scope of this work to settle this debate, however, it should be noted that if demand characteristics are at least partially responsible for higher ratings in the RH synchronous than the asynchronous condition, this could also be true for the moment by moment Ownership ratings provided with the potentiometer. Considering how fundamental this paradigm has become for the investigation of the body representation, future studies should further investigate what is the relative role of multisensory integration processes and hypnotic suggestibility in determining the RHI and whether better control conditions can be developed to measure the illusion.

Summarizing, in the present work we have shown the temporal dynamics of the RHI during visuo-tactile stimulation, and how most of the feeling of ownership is established already during the first 20 s of stimulation. Also, we have shown that, on average, the feeling of ownership disappears within about one minute from the interruption of the visuo-tactile stimulation. Finally, we have proposed a parallel between the RHI and Classical Pavlovian Conditioning and we have suggested that the former should draw inspiration from the latter in investigating how the temporal parameter determines the successfulness of the illusion and the relative contribution of the number, rate, and duration of stimulation.

## Methods

### Participants

Thirty (23 females, 7 males, mean age = 18.96, standard deviation = 0.75) participated in exchange for credit in an introductory psychology course at the University of Virginia. All participants were right-handed and had normal or corrected-to-normal vision.

### Ethics declaration

All participants provided written informed consent before participation in the study. All the experimental protocols were approved by the Institutional Review Board for Social and Behavioural Studies at the University of Virginia. All methods utilized in this study for data collection were carried out following the relevant regulations. In particular, all procedures performed in studies involving human participants were in accordance with the ethical standards of the institutional research committee and with the 1964 Helsinki Declaration and its later amendments.

### Materials

#### Rubber hand and wooden block

The rubber hand was a realistic, life-sized, right-shaped prosthetic hand. The piece of wood was a wooden block, comparable in size to the rubber hand (9 cm × 23 cm × 2 cm), pale and beige, with the outline of a hand-drawn on the surface.

#### Rubber Hand Illusion box and potentiometer

**Figure 10:**
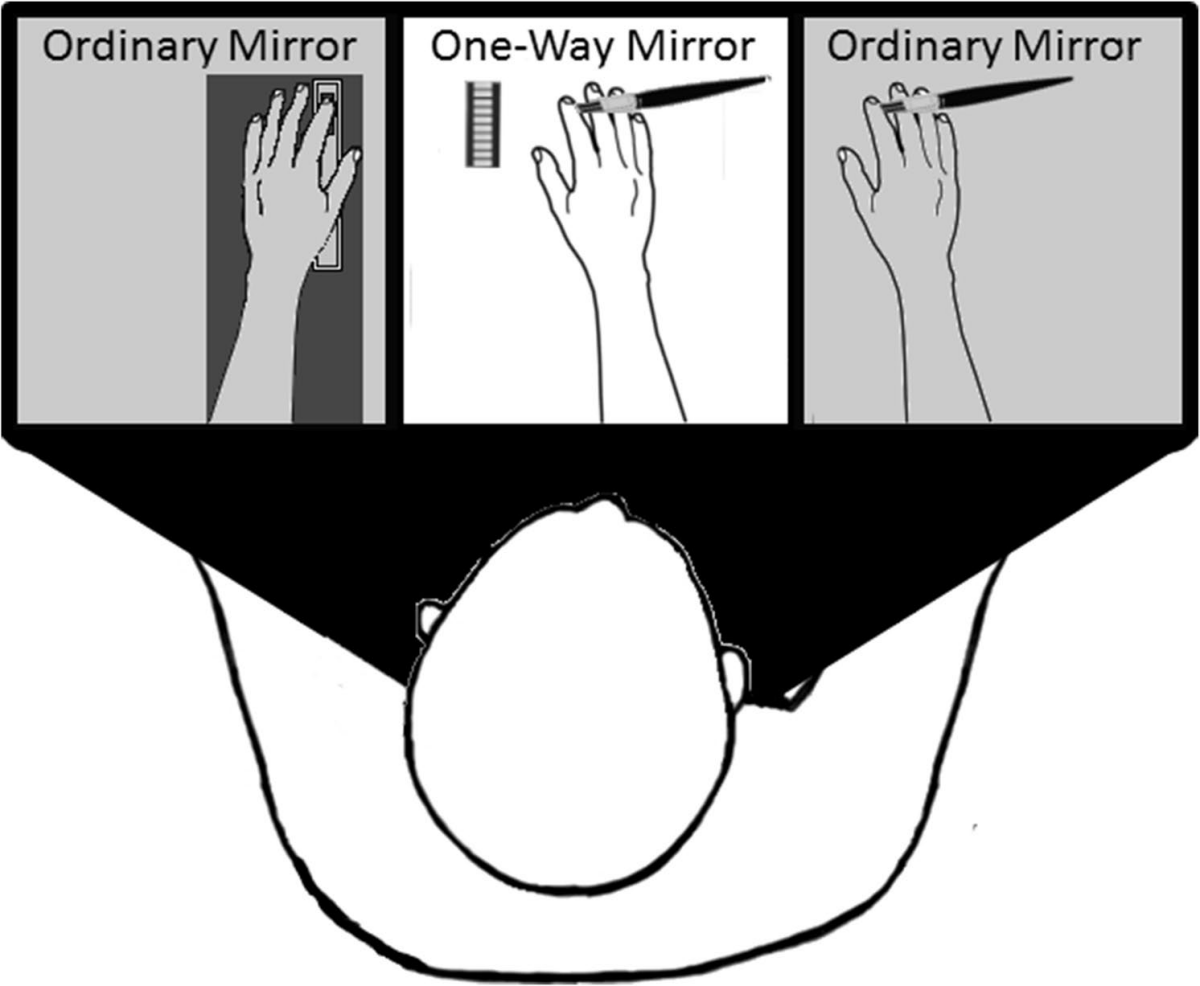
Shows the wooden box used for the Rubber Hand Illusion. The box was divided into three portions with the top covered by a mirror. In the left segment, there was a padded armrest with a linear, slide potentiometer embedded into it. Participants placed their hands in the left and right portions of the box, which was covered by a regular mirror such that the interior of the box was obstructed from view at all times. In the central part, there was a segmented LED bar and the viewed object (either a rubber hand or a piece of wood), and a LED light. When this light was switched on, the interior of the central part of the box became visible to participants. Also depicted in the middle and right boxes are the brushes used to stroke the rubber hand and the participant’s hand.

For the RHI, we used a specially constructed wooden box, measuring 100 cm in width, 40 cm in height, and 20 cm in depth. The box was divided into three segments of equal size, and the viewed object (rubber hand or piece of wood) was positioned inside the central segment aligned to the subject’s midline (see Fig. 10).

In the left segment, there was a padded armrest with a linear, slide potentiometer embedded into it. The potentiometer was 13 cm long and the lever was 1 cm wide. In the middle part of the box, 5 cm to the right of the viewed object, a segmented LED bar, 5 cm long, was placed. This was composed of 10 separate rectangular white LED lights. Both the potentiometer and the LED bar were controlled by an Arduino UNO programmed via Matlab. The potentiometer was programmed to record a value of 0 when the lever’s position was all the way down, and a value of 1000 when it was placed all the way up. The LED bar was used to provide visual feedback to the participants regarding the position of the potentiometer’s lever. The lights were programmed to turn on with an increment of 1 for each 10 points increments of the potentiometer’s values. That is, when the potentiometer was on 0, all the lights were turned off. If the potentiometer was between 1 and 10, the first light would turn on; when it was between 11 and 20, the first two lights would turn on, and so on. The experimenter always made sure that the position of the lever was on 0 at the beginning of each experimental session.

The top of the wooden box was covered by a one-way mirror. The lateral segments of the one-way mirror were obstructed so that the surface always appeared as a regular mirror and participants could not see their hands at any time during the experiment. The central portion of the box contained a LED light that was turned on or off depending on the phase of the experiment. When the light was on, the mirror appeared as a transparent glass, which allowed participants to look at the stimulated object (i.e., rubber hand or piece of wood). When the internal light was off and the light from above was on, the surface appeared as a regular mirror. A ruler with numbers printed in reverse was placed, 45 cm above the mirror, suspended on two poles on the sides of the box. Thus, when the internal light was off, the mirror reflected the numbers of the ruler in their proper orientation and at the same perceived gaze depth as the viewed object.

#### Rubber Hand Illusion questionnaire

We adopted 24 questions from Longo and colleagues^29^ to measure the subjective experience during the RHI (the entire questionnaire is provided in the supplementary materialticular, the questions capture five different components of the experience of the illusion: embodiment of the rubber hand (ten statements, e.g., It seemed like I was looking directly at my own hand, rather than at a rubber hand), loss of the real hand (five statements, e.g., It seemed like I couldn’t really tell where my hand was), movement of the real or rubber hand (three statements, It seemed like my hand was moving towards the rubber hand), deafference of the real hand (three statements, e.g., I had the sensation of pins and needles in my hand.), and affect (three statements, e.g., I found that experience enjoyable). In particular, the Embodiment component has three subcomponents, Ownership, Location and Agency, which refer, respectively, to feelings that the rubber hand belonged to the participant, that the rubber hand and real hand were in the same location, and feelings of being able to move the RH (see^29^). We were particularly interested in the conscious experience of embodiment of the Rubber hand as a measure of the RHI, and the Ownership component as this was the component that most reflected the experience that participants were asked to report with the potentiometer during the stimulation. The other subscales will not be further considered here.

## Procedure

Participants were greeted and informed that they would be asked to report self-perception estimates throughout the experiment. They were then invited to sit at a table and asked to remove any bracelets or rings on their right hand. Then, the experimenter covered the upper body of the participants, from the neck down, with a black cloth and read them the following instructions: “When I turn on the light inside this box, you will be able to see either a rubber hand or a wooden block. I want you to look at this object while I tap your hand and the object with paintbrushes. As soon as you see the object, I want you to use the lever with your left hand to rate whether or not it seems like it is your own hand, rather than a rubber hand or a wooden block. If you feel strongly that the object you are seeing is your own hand, you should push the lever all the way up. If it doesn’t feel like it is your own hand at all, then pull the lever all the way down towards you. Keep moving the lever as the experience changes for you. I will tell you when to stop moving the lever”. The question that participants were answering with the potentiometer was extracted from the “Ownership” scale of the RHI questionnaire (for more details, see materials and procedure).

Afterwards, participants rested their right hand inside the right segment of the box, at a lateral distance of 25.5 cm between the participant’s index finger and the index finger of the rubber hand, and their left hand in the left segment of the box, with the arm resting on the armrest and the index finger placed on the potentiometer’s lever. Then, participants underwent a training phase that allowed them to familiarize themselves with the experimental setup. During this phase, there were no objects (hand or wood) in front of the participant. The experimenter invited participants to move the potentiometer’s lever up and down until they were confident that they had a good feeling of how long the potentiometer was and how the lights of the LED bar changed when moving in response to changes in the position of the lever.

Before each stimulation phase, the light inside the box was turned off and the box was illuminated from above. This made the mirror reflective and the object was hidden from view. Participants were asked to make a proprioceptive judgment of the felt location of their hand: they had to verbally report the number on the ruler that was directly above their right index finger (baseline judgement). They were instructed to make this judgment by projecting a parasagittal line from the tip of the right index finger up to the ruler. The ruler was always shifted to a random position before each new judgment, such that the numbers above the participant’s hand were always different. This ensured that participants could not memorize the number corresponding to the index finger and each judgement was independent of the others. This proprioceptive judgement phase lasted about 10 s.

The baseline judgment was followed by the visuo-tactile stimulation phase (120 s): here the light inside the box was turned on to illuminate the viewed object. This made the central portion of the mirror appear transparent, allowing the participant to see the LED bar and the object, rubber hand, or piece of wood, while the experimenter delivered the visuo-tactile stimulation with the brush. The experimenter manually delivered the tactile stimulation using two identical paintbrushes. In the synchronous stimulation, the experimenter stroked both the participant’s hand and the viewed object at the same time, at a speed of approximately one stroke per second. In the asynchronous stimulation, the experimenter stroked alternatively the participant’s hand and the viewed object. The latency between strokes was between 500 and 1000 ms. Each stimulation period lasted 120 s, the post-stimulation period lasted an additional 120 s. Participants were instructed to use their left hand to provide the Ownership Potentiometer Ratings throughout this stimulation phase (that is, using the level to rate whether or not the viewed object seemed like their own hand).

After the stimulation phase, participants provided a second proprioceptive judgement (post-stimulation judgement).

Then, the light inside the box was switched on, allowing participants to see the object again. Participants were instructed to keep providing the ownership potentiometer ratings until told by the experimenter to stop. During this period no tactile stimulation was delivered to the object. This period lasted 120 s (post-stimulation phase).

At the end of each condition (Rubber Hand Synchronous, Rubber Hand Asynchronous, Wood Synchronous, Wood asynchronous), participants were asked to answer the RHI Questionnaire.

After each questionnaire, participants took a short break during which they were encouraged to rest before moving to the next experimental session and to move their hands to prevent the transfer of illusion across conditions. We did not measure the length of the breaks; however, all participants took at least 30 s approximately between conditions and all participants were encouraged to move their arms before moving to the next section. Moreover, cross-over effects were unlikely given that, at the end of each trial, participants were responding to the RHI questionnaire which took approximately one minute and was administered on paper and pen.

Summarizing, each experimental condition comprised the following phases: proprioceptive judgement (i.e., baseline judgment), visuo-tactile stimulation phase (120 s), second proprioceptive judgement (i.e., post-stimulation judgement), post-stimulation phase (120 s), RHI questionnaire. Participants were required to provide the ownership potentiometer ratings throughout the stimulation and post-stimulation phases. There were four different conditions, depending on the type of visuo-tactile stimulation and the viewed object: rubber hand synchronous, rubber hand asynchronous, wood synchronous, and wood asynchronous. Each participant was tested in all four conditions, with the order counterbalanced between participants using a latin square design.

Previous studies showed that the illusion is successful only when the timing of visuo-tactile stimulation is synchronous (e.g.^6,12,27,62^), and when the viewed object resembles an internal representation of the human body and it’s placed in an anatomically plausible position (e.g.^12,63^). For this reason, we used the asynchronous stimulation and the piece of wood to ensure that higher ratings in the RH synchronous were caused by the multisensory integration of spatiotemporal congruent events and by the embodiment of an anatomically plausible viewed object.

## Analysis and measures

### Statistical approach

The experimental design was a 2 × 2 factorial with two within-subject factors: viewed object (rubber hand versus piece of wood) and timing of visuo-tactile stimulation (synchronous versus asynchronous).

Analyses were performed using estimation statistics based on bootstrapped confidence intervals (CIs) and represented with Cumming estimation plots^64,65^. These plots show the raw data for each condition and the paired difference with 95% bias corrected accelerated confidence interval based on 5000 bootstrap samples. Paired differences across conditions were estimated based on the mean (M_diff_), for normally distributed data, or median (Me_diff_), for non-normally distributed data. The inference was based on the inspection of the estimated difference across conditions and the precision of such estimate (i.e. length of the CI): CIs fully overlapping with 0 were interpreted as indicative of no evidence of effect; CIs not overlapping with 0 were interpreted as indicative of weak, moderate, or strong evidence of effect based on the size of the estimated difference and its precision (the longer the CI, the weaker evidence^64,66^). Data were pre-processed in R. The analyses were computed using the web application available at https://www.estimationstats.com/^65^.

### Dependent measures

#### Ownership potentiometer ratings

Ownership potentiometer ratings were recorded at 1 sample per second during the 240 s of the experiment (stimulation and post-stimulation phases).

Our main interest was to test how the feeling of ownership progresses over time. Hence, we eliminated participants that didn’t report feeling a sensation of ownership over the rubber hand. In particular, a total of 3 participants that never moved the potentiometer above 0 during the total 120 s of stimulation in the Rubber Hand Synchronous condition were removed from the analysis The final sample included 27 participants (22 Females, 5 males; mean age = 19, standard deviation = 0.78).

For about 10 s after the stimulation phase, participants were engaged in providing the proprioceptive judgement and could not report the ownership potentiometer ratings, however, the values from the potentiometer were still recorded. For this reason, the ratings during the first 10 s post-stimulation were removed from further analysis.

#### Proprioceptive drift and questionnaires

Participants provided two proprioceptive judgments, a baseline judgement before the stimulation phase, and a second one afterwards, at the end of the 120 s of stimulation. The difference between these two is referred to as the proprioceptive drift and represents the change in the perceived position of the hand due to the visuo-tactile stimulation.

The RHI questionnaire scorings were averaged following Longo et al.^29^. In particular, we used this questionnaire to calculate Embodiment and Ownership scorings which were used as a proxy of the conscious feeling of the illusion, in line with previous studies from our lab (e.g.,^67–69^) and previous literature^29,30^.

### Analysis

#### Proprioceptive drift and questionnaires

We tested whether, at a group level, participants experienced the RHI as assessed by classic measures (i.e., proprioceptive drift, which is usually considered as a measure of the implicit component of the illusion, and rubber hand questionnaires, which measure the explicit component of the illusion). These analyses are reported in the supplementary materials.

#### Ownership potentiometer ratings

##### Stimulation phase

The first analysis aimed at testing whether, during the visuo-tactile stimulation, participants reported higher feelings of ownership over the rubber hand synchronous as compared to all other experimental conditions. To test this, first, we averaged the ownership potentiometer ratings during the 120 s of stimulation in the four different conditions, and then we compared the scorings in the different conditions using multiple paired estimation statistics.

##### Post-stimulation phase

The second analysis aimed at testing whether the illusion was present during the post-stimulation phase. For this analysis, we averaged ownership potentiometer ratings during the 120 s after the visuo-tactile stimulation in the four different conditions. Then, we again used multiple paired estimation statistics to test whether ownership scores were higher in the Rubber Hand Synchronous as compared to all other experimental conditions.

##### Progression over time during stimulation

The third and fourth analyses aimed at investigating the progression over time of the sensation of ownership. Therefore, these analyses only focused on the RH Synchronous versus RH Asynchronous conditions.

In particular, the third analysis aimed at investigating the onset time of the sensation of ownership. To this aim, ownership potentiometer ratings for the 120 s of stimulation were averaged in 4 blocks, 30 s each, and for each experimental condition. We used multiple paired estimation statistics to test whether there was a difference between the Rubber Hand Synchronous and the Rubber hand Asynchronous conditions for each time block.

##### Progression over time post-stimulation

The fourth analysis aimed at testing the fading time of the sensation of ownership, that is, when, on average, participants stopped reporting a feeling of owning the RH. To this aim, sensation scores for the 120 s of the post-stimulation phase were averaged in 4 different 30 s blocks for each experimental condition. As for the previous analysis, we used multiple paired estimation statistics to test whether there was a difference between the RH Synchronous and the RH Asynchronous conditions for each time block.

##### Changepoint analysis

One of the advantages of having a continuous measure of the feeling of Ownership is that we can treat participants’ ratings as a continuous signal. To determine the onset and fading times of the feeling of ownership, we investigated where the Ownership Potentiometer Ratings change most abruptly. In particular, first, we averaged the Ownership Potentiometer Ratings across all participants in the RH synchronous and RH asynchronous conditions. The RHI is usually quantified as the difference in illusory feelings between the RH synchronous and the RH asynchronous. Accordingly, to obtain a measure of the illusory effect of Ownership generated by the synchronous stimulation, we subtracted the Ownership Potentiometer Ratings in the RH asynchronous from the RH synchronous ratings. For clarity, we will refer to this as the Ownership Effect. To ensure that the changepoints analysis would not be biased by small transient changes in the signal, we smoothed the Ownership Effect signal by applying a moving average filter with a five-seconds window. As a next step, we divided the signal into two parts: 120 s of stimulation phase and 120 s post-stimulation phase. Finally, we used the *findchangepts* function from Matlab to find the points where the signal changed most abruptly with two additional options: (1) a “linear” statistic and (2) two maximum points. A changepoint is defined as the point in time where a statistical property changes significantly. To find a signal changepoint, *findchangepts* employs a parametric global method. The function: (1) Chooses a point and divides the signal into two sections. (2) Computes an empirical estimate of the desired statistical property for each section. (3) At each point within a section, measures how much this property deviates from the empirical estimate. Adds the deviations for all points. (4) Adds the deviations section-to-section to find the total residual error. (5) Varies the location of the division point until the total residual error attains a minimum. When specifying to use a linear statistic, as we did, the function finds the points where the mean and the slope of the signal change most abruptly. This is accomplished by using as total deviation the sum of squared differences between the signal values and the predictions of the least-squares linear fit through the values. This quantity is also known as the *error sum of squares*, or *SSE*.

The limit of two points was decided in a trial-and-error fashion: visual inspection showed that adding more changepoints resulted in segments with a smaller slope that seem to indicate a negligible change in the signal.

We repeated the changepoint analysis twice: once for the 120 s of visuo-tactile stimulation (we will refer to these as “onset changepoints”) and once for the 120 s post-stimulation (we will refer to these as “fading changepoints”).

After finding these points in the signal, we tested whether the average Ownership Potentiometer Ratings differed between the RH Synchronous and the RH Asynchronous conditions at the first changepoint during the stimulation and the last changepoint post-stimulation. This analysis aimed at investigating whether the Illusory feeling of Ownership was already established and disappeared at the respective points in time.

##### Individual changepoints

Next, we tested whether there was a relationship between how fast the Ownership Effect increased and how fast it decreased. To this aim, first, as in the previous analysis, we used the *findchangepts function* on the Ownership Effect, once for the visuo-tactile stimulation period and once for the post-stimulation. However, this time we applied the *findchangepts* function for each individual separately. For two participants, no changepoint could be found given that the Ownership Effect signal was stationary; these were not included in these analyses. Then, we detected and removed outlier values using the R *boxplot* function, which classifies outliers based on the interquartile criterion. For each analysis, we report the number of participants included after the outliers were removed. We ran four separate Spearman correlations with bootstrapped confidence intervals to investigate the relationship between the first of the two Onset Changepoints and: (1) the first of the two Fading Changepoints; (2) the average Ownership Effect during the visuo-tactile stimulation; (3) the Embodiment scores as measured via questionnaires; (4) the proprioceptive drifts.

##### Relation to classical measures of illusion

To test whether there was a relationship between ownership potentiometer ratings and classic measures of explicit illusion (i.e., questionnaires), we ran a Spearman correlation between the Ownership Potentiometer Ratings averaged during the 120 s of RH synchronous visuo-tactile stimulation and the Ownership component of the illusion as measured with the questionnaires. It is important to notice that the RHI questionnaires are administered at the end of each trial and they ask participants to rate their feelings during the stimulation. It is reasonable to expect that participants rate the experience based on the time-period when the illusory feeling was at its peak, rather than by averaging what they were feeling throughout the entire stimulation. For this reason, we ran an additional analysis comparing the Ownership Potentiometer Ratings in the RH synchronous during the last 30 s of visuo-tactile stimulation (seconds 90 to 120) with the questionnaire Ownership ratings in the same condition.

## Supplementary Information

Supplementary Information.

## Data Availability

All the data collected for this experiment are available online on Open Science Framework at: https://osf.io/t92es/files/.

## References

[1] Barsalou LW (2010). Grounded cognition: Past, present, and future. Top. Cogn. Sci..

[2] de Vignemont F, Alsmith A (2018). The Subject’s Matter.

[3] Proffitt D, Baer D (2020). Perception: How Our Bodies Shape Our Minds.

[4] Botvinick M, Cohen J (1998). Rubber hands ‘feel’ touch that eyes see. Nature.

[5] Ehrsson HH, Stein BE (2012). The concept of body ownership and its relation to multisensory integration. The New Handbook of Multisensory Processes.

[6] Tsakiris M, Haggard P (2005). The Rubber Hand Illusion revisited: Visuotactile integration and self-attribution. J. Exp. Psychol. Hum. Percept. Perform..

[7] Apps MAJ, Tsakiris M (2013). The free-energy self: A predictive coding account of self-recognition. Neurosci. Biobehav. Rev..

[8] Friston K (2005). A theory of cortical responses. Philos. Trans. R. Soc. B Biol. Sci..

[9] Friston KJ, Stephan KE (2007). Free-Energy and the Brain.

[10] Limanowski J (2013). Minimal self-models and the free energy principle minimal self-models and the free energy principle. Philos. Trans. R. Soc. B Biol. Sci..

[11] Zeller D, Litvak V, Friston KJ, Classen J (2013). Sensory processing and the Rubber Hand Illusion: An evoked potentials study. J. Cogn. Neurosci..

[12] Costantini M, Haggard P (2007). The Rubber Hand Illusion: Sensitivity and reference frame for body ownership. Conscious. Cogn..

[13] Farnè A, Làdavas E (2000). Dynamic size-change of hand peripersonal space following tool use. NeuroReport.

[14] Maravita A, Spence C, Kennett S, Driver J (2002). Tool-use changes multimodal spatial interactions between vision and touch in normal humans. Cognition.

[15] Ferri F (2013). Upcoming tactile events and body ownership in schizophrenia. Schizophr. Res..

[16] Peled A, Ritsner M, Hirschmann S, Geva AB, Modai I (2000). Touch feel illusion in schizophrenic patients. Biol. Psychiatry.

[17] Cascio CJ, Foss-Feig JH, Burnette CP, Heacock JL, Cosby AA (2012). The Rubber Hand Illusion in children with autism spectrum disorders: delayed influence of combined tactile and visual input on proprioception. Autism.

[18] Kalckert A, Ehrsson H (2017). The onset time of the ownership sensation in the moving Rubber Hand Illusion. Front. Psychol..

[19] Perepelkina O, Vorobeva V, Melnikova O, Arina G, Nikolaeva V (2018). Artificial hand illusions dynamics: Onset and fading of static rubber and virtual moving hand illusions. Conscious. Cogn..

[20] Ehrsson HH, Wiech K, Weiskopf N, Dolan RJ, Passingham RE (2007). Threatening a rubber hand that you feel is yours elicits a cortical anxiety response. Proc. Natl. Acad. Sci. USA..

[21] Lloyd DM (2007). Spatial limits on referred touch to an alien limb may reflect boundaries of visuo-tactile peripersonal space surrounding the hand. Brain Cogn..

[22] Abdulkarim Z, Hayatou Z, Ehrsson HH (2021). Sustained Rubber Hand Illusion after the end of visuotactile stimulation with a similar time course for the reduction of subjective ownership and proprioceptive drift. Exp. Brain Res..

[23] Reader AT, Trifonova VS, Ehrsson HH (2021). The relationship between referral of touch and the feeling of ownership in the Rubber Hand Illusion. Exp. Brain Res..

[24] Motyka P, Litwin P (2019). Proprioceptive precision and degree of visuo-proprioceptive discrepancy do not influence the strength of the Rubber Hand Illusion. Perception.

[25] Reader AT, Trifonova VS, Ehrsson HH (2021). Little evidence for an effect of the Rubber Hand Illusion on basic movement. Eur. J. Neurosci..

[26] Watson R, Pavani F, De Gelder B (2017). Affective vocalizations influence body ownership as measured in the Rubber Hand Illusion. PLoS ONE.

[27] Ehrsson HH, Spence C, Passingham RE (2004). That’s my hand! Activity in premotor cortex reflects feeling of ownership of a limb. Science.

[28] Garofalo S, Giovagnoli S, Orsoni M, Starita F, Benassi M (2022). Interaction effect: Are you doing the right thing?. PLoS ONE.

[29] Longo MR, Schüür F, Kammers MPM, Tsakiris M, Haggard P (2008). What is embodiment? A psychometric approach. Cognition.

[30] Romano D, Maravita A, Perugini M (2021). Psychometric properties of the embodiment scale for the Rubber Hand Illusion and its relation with individual differences. Sci. Rep..

[31] Costantini M (2016). Temporal limits on Rubber Hand Illusion reflect individuals ’ temporal resolution in multisensory perception. Cognition.

[32] Burin D (2019). Relationships between personality features and the Rubber Hand Illusion: An exploratory study. Front. Psychol..

[33] Cutts SA, Fragaszy DM, Mangalam M (2019). Consistent inter-individual differences in susceptibility to bodily illusions. Conscious. Cogn..

[34] Peled A, Pressman A, Geva AB, Modai I (2003). Somatosensory evoked potentials during a rubber-hand illusion in schizophrenia. Schizophr. Res..

[35] Thakkar KN, Nichols HS, McIntosh LG, Park S (2011). Disturbances in body ownership in schizophrenia: Evidence from the Rubber Hand Illusion and case study of a spontaneous out-of-body experience. PLoS ONE.

[36] Germine L, Benson TL, Cohen F, Hooker CI (2013). Psychosis-proneness and the Rubber Hand Illusion of body ownership. Psychiatry Res..

[37] Galigani M, Fossataro C, Gindri P, Conson M, Garbarini F (2022). Monochannel preference in autism spectrum conditions revealed by a non-visual variant of Rubber Hand Illusion. J. Autism Dev. Disord..

[38] Brunel L, Carvalho PF, Goldstone RL (2015). It does belong together: Cross-modal correspondences influence cross-modal integration during perceptual learning. Front. Psychol..

[39] Mitchel AD, Weiss DJ (2011). Learning across senses: Cross-modal effects in multisensory statistical learning. J. Exp. Psychol. Learn. Mem. Cogn..

[40] Lauzon S, Abraham AE, Curcin K, Butler BE, Stevenson RA (2022). The relationship between multisensory associative learning and multisensory integration. Neuropsychologia.

[41] Clark R (2004). The classical origins of Pavlov’s conditioning. Integr. Physiol. Behav. Sci..

[42] Pavlov IP (1927). Conditioned Reflexes: An Investigation of the Physiological Activity of the Cerebral Cortex.

[43] Lin JY, Arthurs J, Reilly S (2017). Conditioned taste aversions: From poisons to pain to drugs of abuse. Psychon. Bull. Rev..

[44] Ohman A, Eriksson A, Olofsson C (1975). One-trial learning and superior resistance to extinction of autonomic responses conditioned to potentially phobic stimuli. J. Comp. Physiol. Psychol..

[45] Garcia J, Koelling RA (1966). Relation of cue to consequence in avoidance learning. Psychon. Sci..

[46] Rao IS, Kayser C (2017). Neurophysiological correlates of the Rubber Hand Illusion in late evoked and alpha/beta band activity. Front. Hum. Neurosci..

[47] Dummer T, Picot-annand A, Neal T, Moore C (2014). Movement and the Rubber Hand Illusion. Perception.

[48] Kalckert A, Ehrsson HH (2014). The moving Rubber Hand Illusion revisited: Comparing movements and visuotactile stimulation to induce illusory ownership. Conscious. Cogn..

[49] De Beir, A. *et al.* Developing new frontiers in the Rubber Hand Illusion: Design of an open source robotic hand to better understand prosthetics. in *IEEE RO-MAN 2014—23rd IEEE International Symposium on Robot and Human Interactive Communication: Human-Robot Co-Existence: Adaptive Interfaces and Systems for Daily Life, Therapy, Assistance and Socially Engaging Interactions*, 905–910. 10.1109/ROMAN.2014.6926368 (2014).

[50] Salagean A, Hadnett-Hunter J, Finnegan DJ, De Sousa AA, Proulx MJ (2022). A virtual reality application of the Rubber Hand Illusion induced by ultrasonic mid-air haptic stimulation. ACM Trans. Appl. Percept..

[51] Ariza, O. *et al.* Inducing body-transfer illusions in VR by providing brief phases of visual-tactile stimulation. in *SUI 2016—Proceedings of the 2016 Symposium on Spatial User Interaction*, 61–68. 10.1145/2983310.2985760 (2016).

[52] Azanõn E (2016). Multimodal contributions to body representation. Multisens. Res..

[53] Martel M (2016). Tool-use: An open window into body representation and its plasticity. Cogn. Neuropsychol..

[54] Christ O (2012). The Rubber Hand Illusion: Maintaining factors and a new perspective in rehabilitation and biomedical engineering?. Biomed. Tech..

[55] Lush P (2020). Demand characteristics confound the Rubber Hand Illusion. Collabra Psychol..

[56] Lush P, Seth AK, Dienes Z (2021). Hypothesis awareness confounds asynchronous control conditions in indirect measures of the Rubber Hand Illusion. R. Soc. Open Sci..

[57] Roseboom W, Lush P (2022). Serious problems with interpreting rubber hand “illusion” experiments. Collabra Psychol..

[58] Lush P (2020). Trait phenomenological control predicts experience of mirror synaesthesia and the Rubber Hand Illusion. Nat. Commun..

[59] Ehrsson HH, Fotopoulou A, Radziun D, Longo MR, Tsakiris M (2022). No specific relationship between hypnotic suggestibility and the Rubber Hand Illusion. Nat. Commun..

[60] Slater M, Ehrsson HH (2022). Multisensory integration dominates hypnotisability and expectations in the Rubber Hand Illusion. Front. Hum. Neurosci..

[61] Reader AT (2022). What do participants expect to experience in the Rubber Hand Illusion? A conceptual replication of lush (2020). Collabra Psychol..

[62] Shimada S, Fukuda K, Hiraki K (2009). Rubber Hand Illusion under delayed visual feedback. PLoS ONE.

[63] Haans A, Ijsselsteijn WA, de Kort YAW (2008). The effect of similarities in skin texture and hand shape on perceived ownership of a fake limb. Body Image.

[64] Cumming G (2014). The new statistics: Why and how. Psychol. Sci..

[65] Ho J, Tumkaya T, Aryal S, Choi H, Claridge-Chang A (2019). Moving beyond P values: Data analysis with estimation graphics. Nat. Methods.

[66] Calin-Jageman RJ, Cumming G (2019). The new statistics for better science: Ask how much, how uncertain, and what else is known. Am. Stat..

[67] Finotti G, Costantini M (2016). Multisensory body representation in autoimmune diseases. Sci. Rep..

[68] Weser V, Finotti G, Costantini M, Pro DR (2012). Multisensory integration induces body ownership of a handtool, but not any handtool. New Handb. Multisens. Process..

[69] Finotti G, Migliorati D, Costantini M (2018). Multisensory integration, body representation and hyperactivity of the immune system. Conscious. Cogn..

