# Supplementary material for: Temporal dynamics of the Rubber Hand Illusion
Source: Sci Rep. 2023 May 9;13:7526. doi: 10.1038/s41598-023-33747-2 (PMC10170134; doi:10.1038/s41598-023-33747-2)
Supplement: Supplementary file 1 — Supplementary Information. [file 41598_2023_33747_MOESM1_ESM.docx]

# **Temporal dynamics of the Rubber Hand Illusion**

# **Supplementary materials**

Gianluca Finotti^1,2*^, Sara Garofalo^2^, Marcello Costantini ^3^, Dennis R. Proffitt^1^

^1^ Department of Psychology, University of Virginia, Charlottesville, Virginia, USA.

^2^ Center for Studies and Research in Cognitive Neuroscience, Department of Psychology, University of Bologna, Cesena, Italy

^3^ Department of Psychological, Health and Territorial Sciences, University G. d’Annunzio, Chieti-Pescara, Italy

Corresponding Author

*Gianluca Finotti, Center for Studies and Research in Cognitive Neuroscience, Department of Psychology, University of Bologna, Via Rasi e Spinelli 176, 47521, Cesena (FC), Italy, Email address: g.finotti@unibo.it

Here we report additional analyses conducted on classic measures of the Rubber Hand Illusion (RHI), i.e., proprioceptive drifts and RHI questionnaires.

# Results

## Proprioceptive drift

Proprioceptive drift values for two participants were missing due to experimenter’s error (N = 25). With the rubber hand, there was a moderate difference (M_diff_ = 1.68; 95% CI [0.4, 2.88]) between synchronous (M = 1.76, SD = 3.22) and asynchronous (M = 0.08, SD = 1.89) stimulation (see Fig S1). With the wooden bock, there was no difference (M_diff_ = 0.1; 95% CI [-1.72, 1.84]) between synchronous (M = 0.84, SD = 3.91) and asynchronous (M = 0.84, SD = 2.08) stimulation (see Fig S1).

Bar plots show that the proprioceptive drifts were higher in the rubber hand synchronous than in all other experimental conditions (see Fig S2). Summarizing, the analysis of the proprioceptive drifts shows that, on average, after synchronous visuo-tactile stimulation with the RH synchronous, participants tended to perceive the position of their stimulated hand closer to the RH as compared to the pre-stimulation judgement. In line with previous studies (e.g., ^1^) this can be interpreted as evidence that the RHI was successfully elicited on an implicit level.


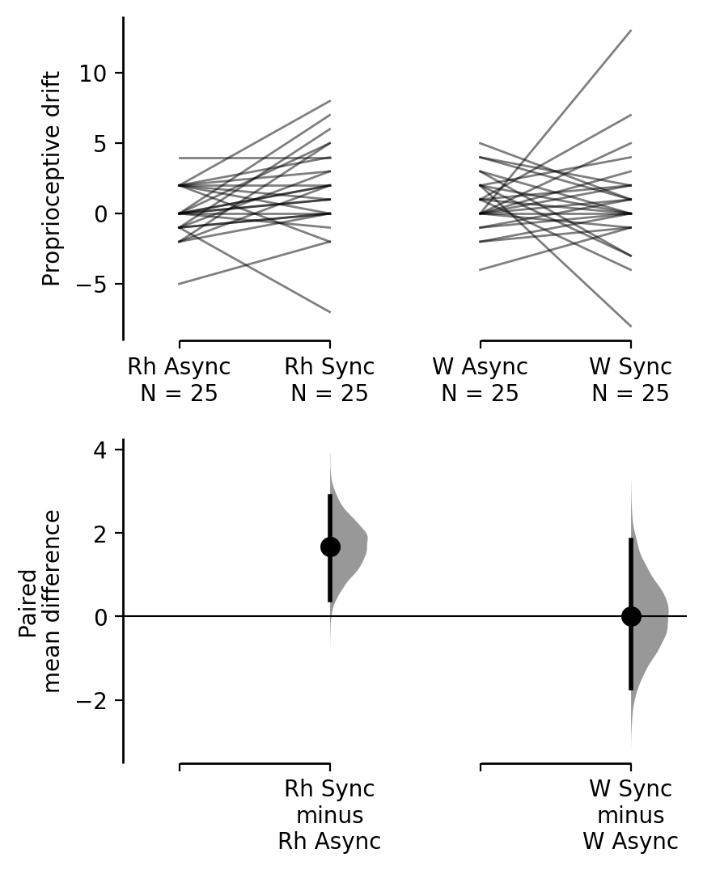


Fig S1 the upper axes show the raw data, that is, the proprioceptive drift scores averaged in the four different conditions, each paired set of observations is connected by a line. On the lower axes, the paired mean difference between RH Synchronous and RH Asynchronous, and between Wood Synchronous and Wood Asynchronous is plotted as a bootstrap sampling distribution. Mean differences are depicted as dots; 95% confidence intervals are indicated by the ends of the vertical error bars.


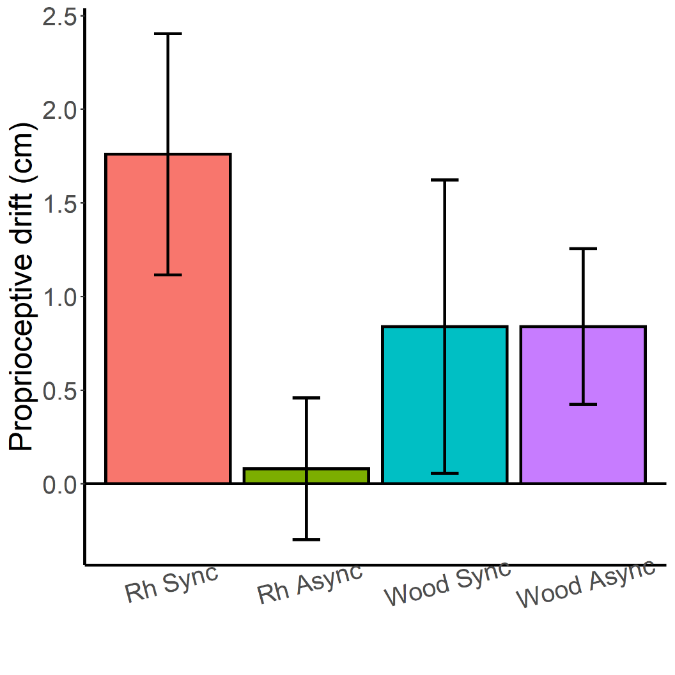


Fig S2 represents the mean and standard error (error bars) for the proprioceptive drift in the four different experimental conditions.

## Questionnaires

For reference on how we calculated the scorings of the RHI questionnaire, see ^2^.

### Embodiment

Embodiment refers to the feeling that the rubber hand belongs to the participant’s body.

With the rubber hand, there was a moderate difference (Me_diff_ = 1.2; 95% CI [1.2, 1.2]) between synchronous (Me = 0.3, IQR = 1.6) and asynchronous (Me = -0.8, IQR = 3.05) stimulation (see Fig S3). With the wooden hand, there was no difference (Me_diff_ = 0.1; 95% CI [0, 0.8]) between synchronous (Me = -1.3, IQR = 2.6) and asynchronous (Me = -2.4, IQR = 2.1) stimulation (see Fig S3).

A raincloud plot shows that the Embodiment scores were higher in the rubber hand synchronous than in all other experimental conditions (see Fig S4), which can be considered as evidence that in this condition the conscious illusory experience that the RH belonged to the participant’s body was successfully induced.


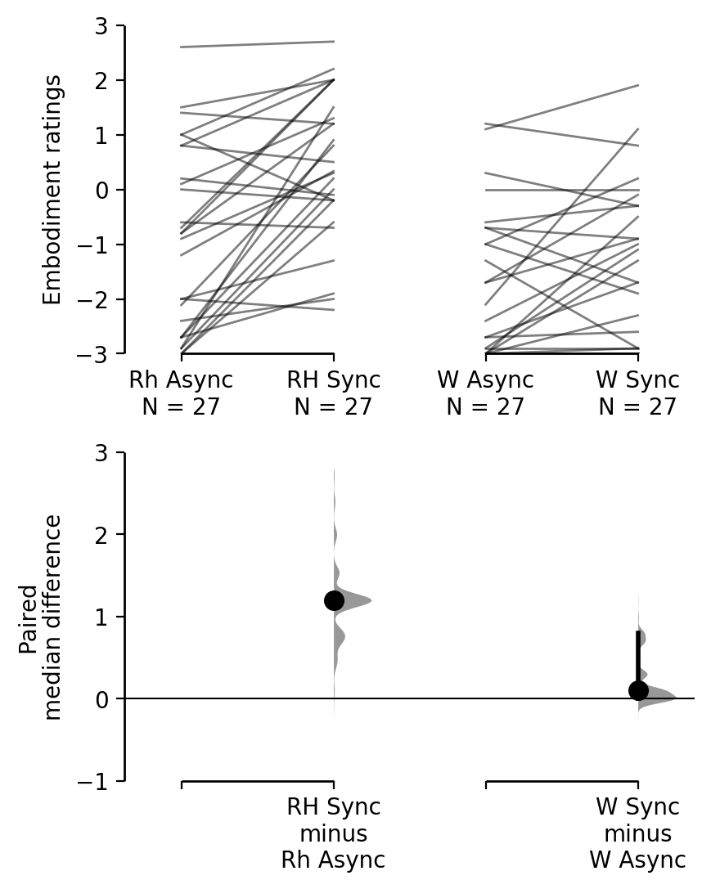


Fig S3 the upper axes show the raw data, that is, the Embodiment component of the illusion as measured by the rubber hand illusion questionnaire. On the lower axes, the paired median difference between RH Synchronous and RH Asynchronous, and between Wood Synchronous and Wood Asynchronous is plotted as a bootstrap sampling distribution. Median differences are depicted as dots; 95% confidence intervals are indicated by the ends of the vertical error bars.


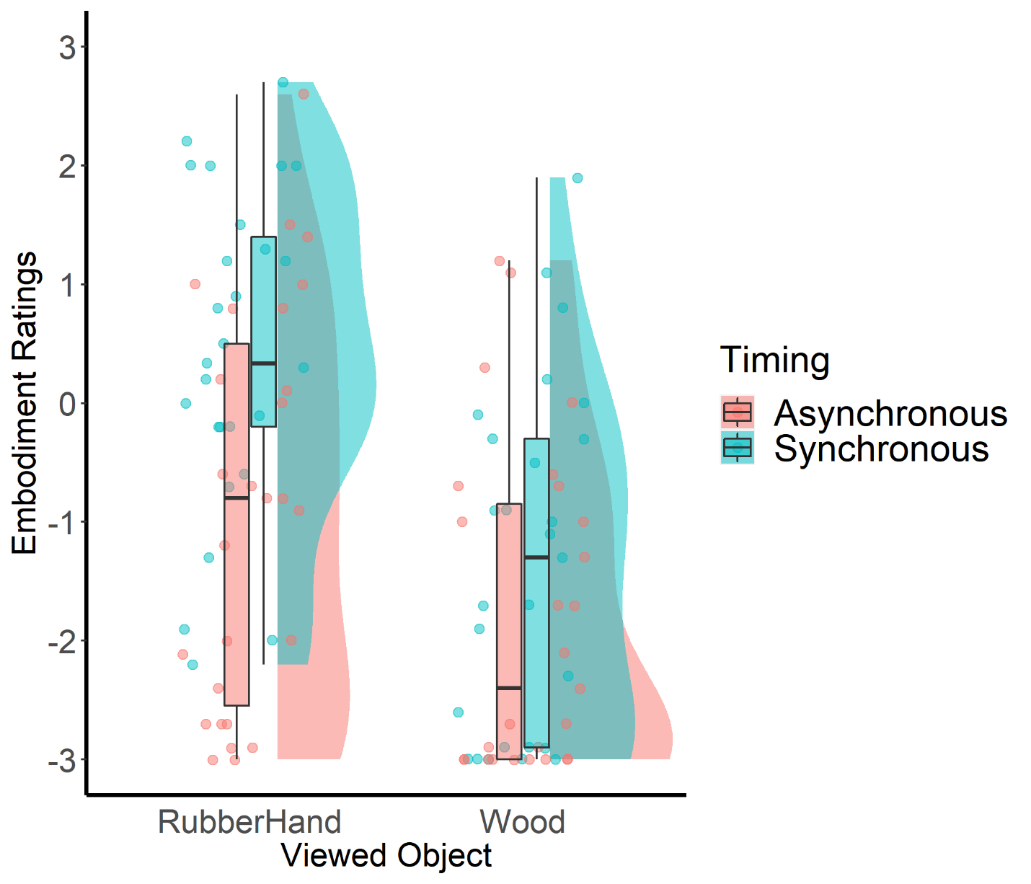


Fig S4 A raincloud plot shows Embodiment scorings as measured with the rubber hand illusion questionnaire for the four different visuo-tactile stimulations.

### Ownership

Ownership is related to the feeling that the RH was part of one’s body and that participants were looking directly at their hand.

With the rubber hand, there was a weak difference (Me_diff_ = 1.2; 95% CI [0, 1.95]) between synchronous (Me = 0.75, IQR = 1.6) and asynchronous (Me = -0.8, IQR = 3.3) stimulation, indicating a probable effect of stimulation in this condition (see Fig S5). With the wooden hand, there was no difference (Me_diff_ = 0; 95% CI [0, 0]) between synchronous (Me = -2, IQR = 3) and asynchronous (Me = -2.8, IQR = 2.1) stimulation (see Fig S5).

A raincloud plot shows that the Ownership scores were higher in the rubber hand synchronous than in all other experimental conditions (see Fig S6).


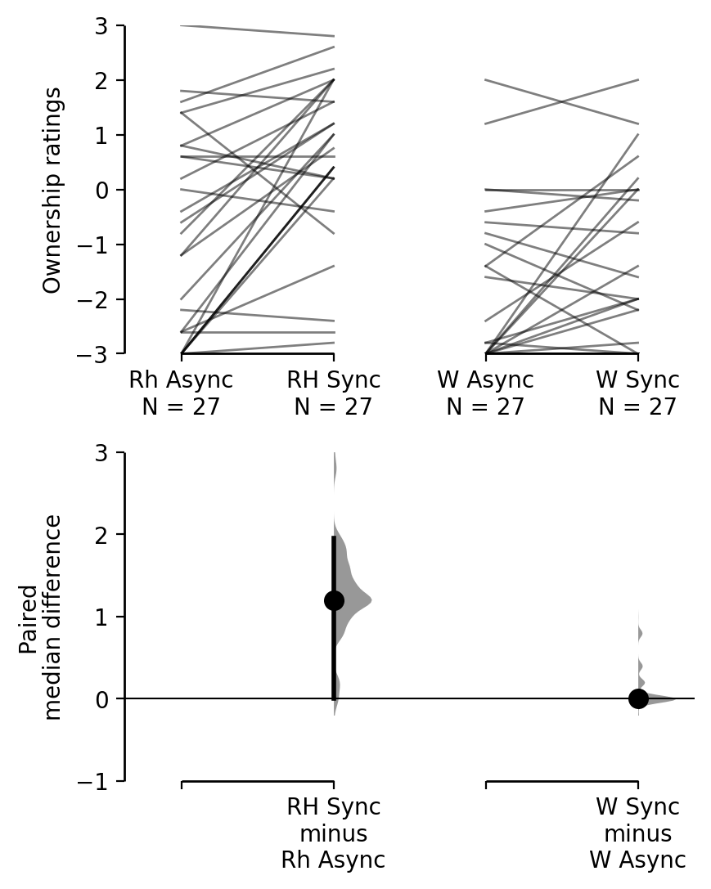


Fig S5 the upper axes show the raw data, that is, the Ownership component of the illusion as measured by the rubber hand illusion questionnaire. On the lower axes, the paired median difference between RH Synchronous and RH Asynchronous, and between Wood Synchronous and Wood Asynchronous is plotted as a bootstrap sampling distribution. Median differences are depicted as dots; 95% confidence intervals are indicated by the ends of the vertical error bars.


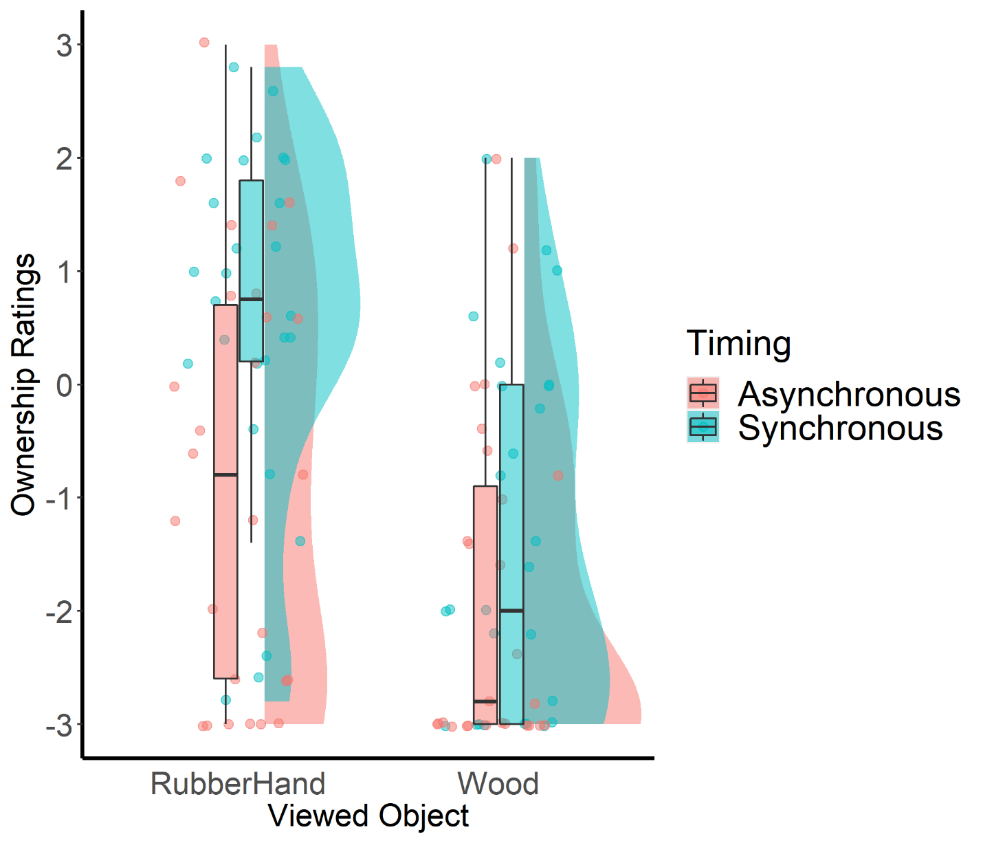


Fig S6 A raincloud plot shows Ownership scorings as measured with the rubber hand illusion questionnaire for the four different visuo-tactile stimulations.

### Loss

Loss is related to the feeling of being unable to move one’s hand and feeling one’s hand disappearing.

With the rubber hand, there was no difference (Me_diff_ = 0.5; 95% CI [-0.16, 0.5]) between synchronous (Me = 0.5, IQR = 1.25) and asynchronous (Me = 0, IQR = 1.83) stimulation (see Fig S7).

With the wooden hand, there was no difference (Me_diff_ = 0.167; 95% CI [0.333, 0.167]) between synchronous (Me = -0.16, IQR = 2) and asynchronous (Me = -0.66, IQR = 1.91) stimulation (see Fig S7).

A raincloud plot shows no difference in Loss ratings in the different conditions (see Fig S8).


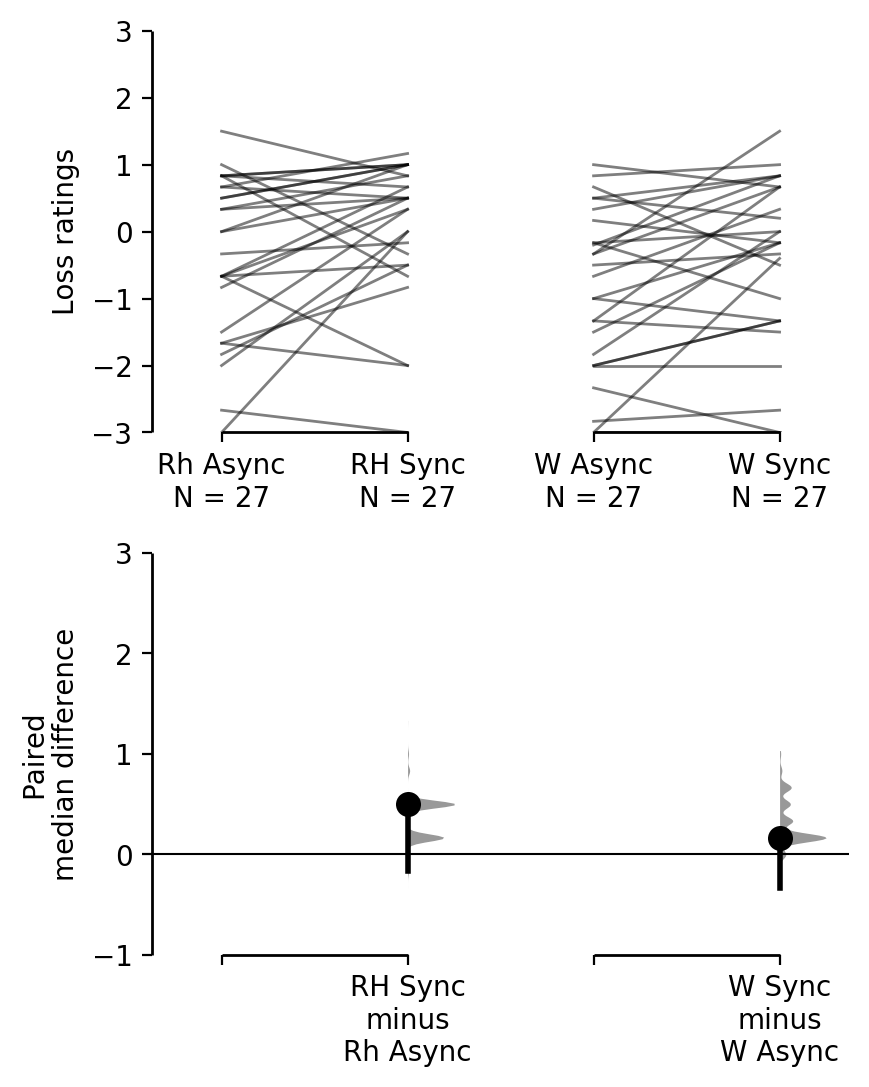


Fig S7 the upper axes show the raw data, that is, the Loss component of the illusion as measured by the rubber hand illusion questionnaire. On the lower axes, the paired median difference between RH Synchronous and RH Asynchronous, and between Wood Synchronous and Wood Asynchronous is plotted as a bootstrap sampling distribution. Median differences are depicted as dots; 95% confidence intervals are indicated by the ends of the vertical error bars.


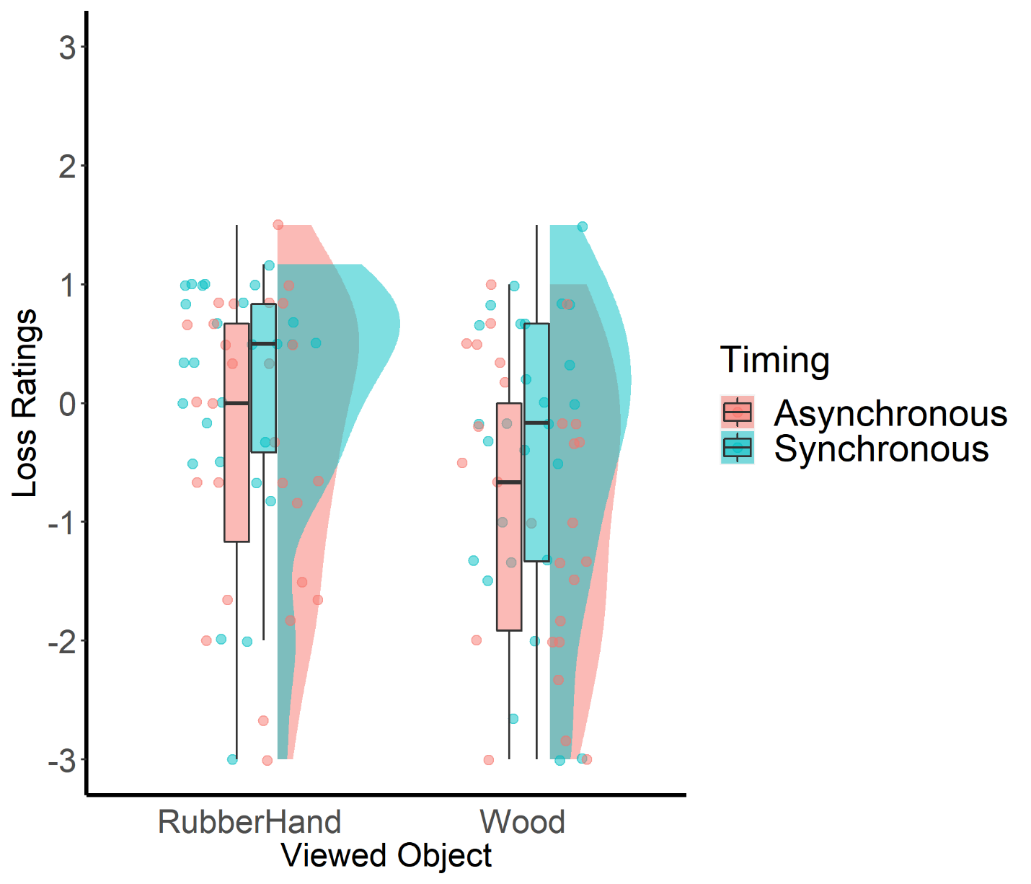


Fig S8 A raincloud plot shows Loss scorings as measured with the rubber hand illusion questionnaire for the four different visuo-tactile stimulations.

### Movement

Movement refers to the perceived motion of one’s hand.

With the rubber hand, there was no difference (Me_diff_ = 0.33; 95% CI [0.0, 0.33]) between synchronous (Me = -1, IQR = 2) and asynchronous (Me = -1.33, IQR = 2.5) stimulation (see Fig S9).

With the wooden hand, there was no difference (Me_diff_ = 0.33; 95% CI [0, 0.66]) between synchronous (Me = -1.66, IQR = 2.5) and asynchronous (Me = -2.66, IQR = 1.66) stimulation (see Fig S9).

A raincloud plot shows that the Movement scores were not different between the rubber hand synchronous and all other experimental conditions (see Fig S10).


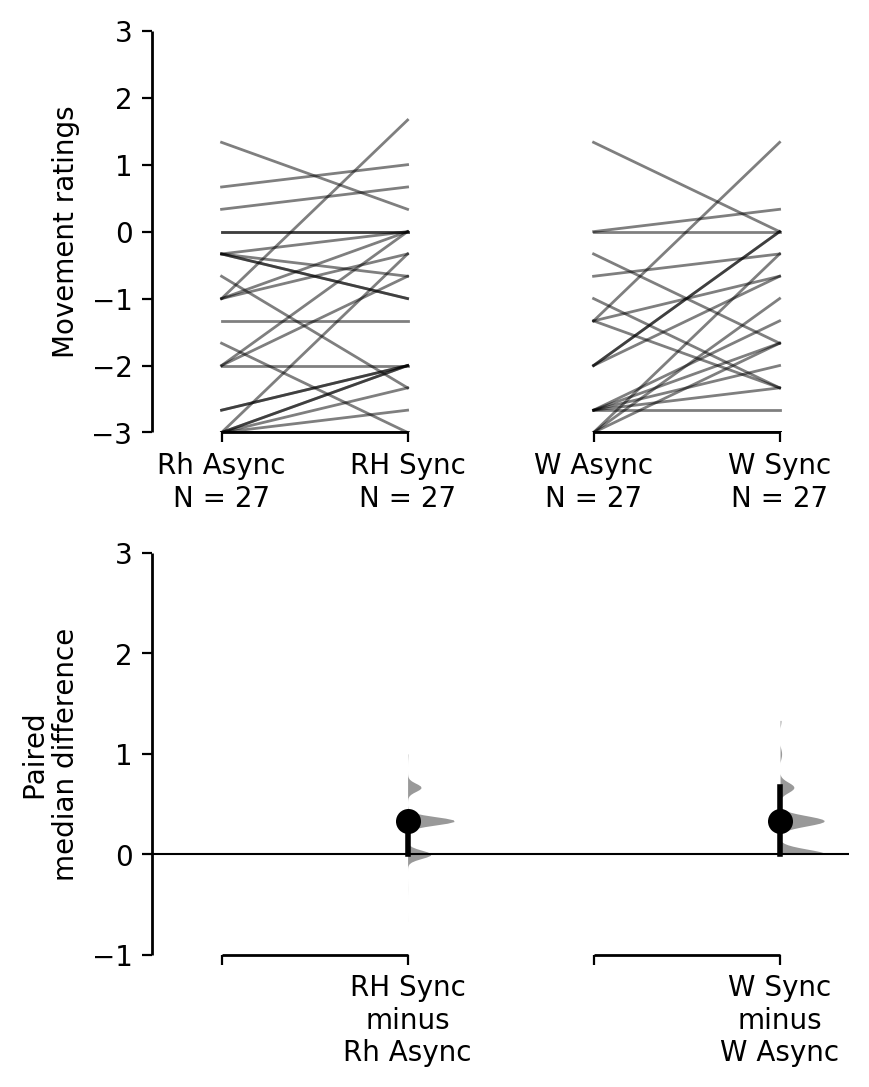


Fig S9 the upper axes show the raw data, that is, the Movement component of the illusion as measured by the rubber hand illusion questionnaire. On the lower axes, the paired median difference between RH Synchronous and RH Asynchronous, and between Wood Synchronous and Wood Asynchronous is plotted as a bootstrap sampling distribution. Median differences are depicted as dots; 95% confidence intervals are indicated by the ends of the vertical error bars.


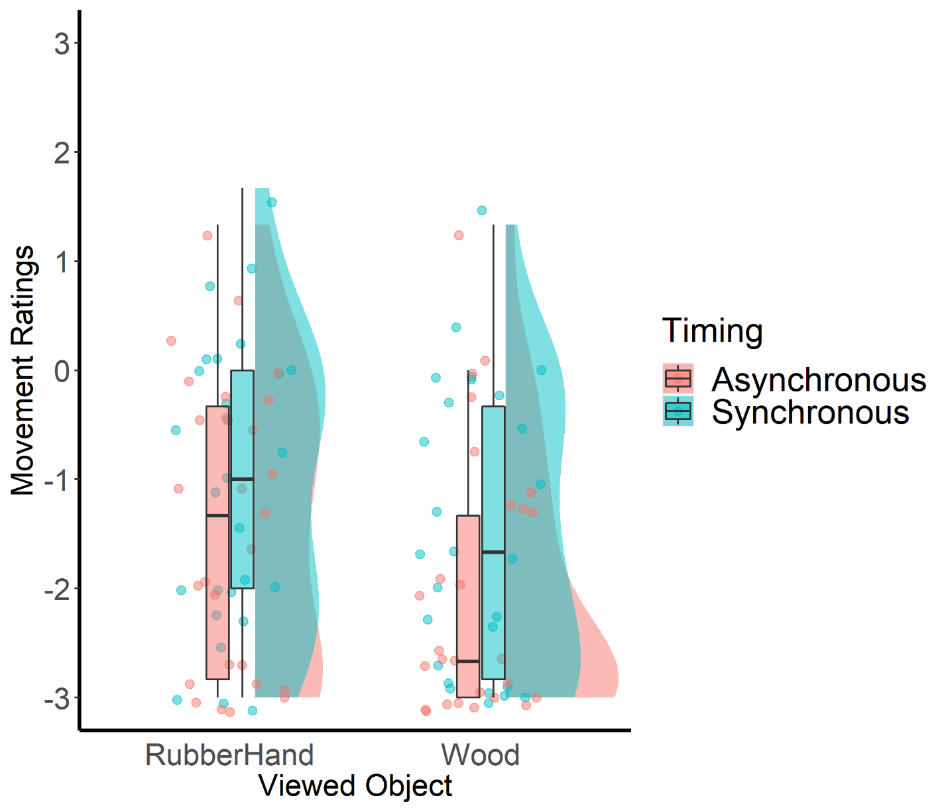


Fig S10 A raincloud plot shows Movement scorings as measured with the rubber hand illusion questionnaire for the four different visuo-tactile stimulations.

### Affect

Affect refers to the experience being enjoyable and interesting.

With the rubber hand, there was no difference (Me_diff_ = 0.33; 95% CI [0.0, 0.66]) between synchronous (Me = -1.66, IQR = 1) and asynchronous (Me = 1, IQR = 1.66) stimulation (see Fig S11).

With the wooden hand, there was no difference (Me_diff_ = 0; 95% CI [0, 0]) between synchronous (Me = 1, IQR = 1) and asynchronous (Me = 1, IQR = 2) stimulation (see Fig S11).

A raincloud plot shows that the Affect scores were similar in the rubber hand synchronous and in all other experimental conditions (see Fig S12).


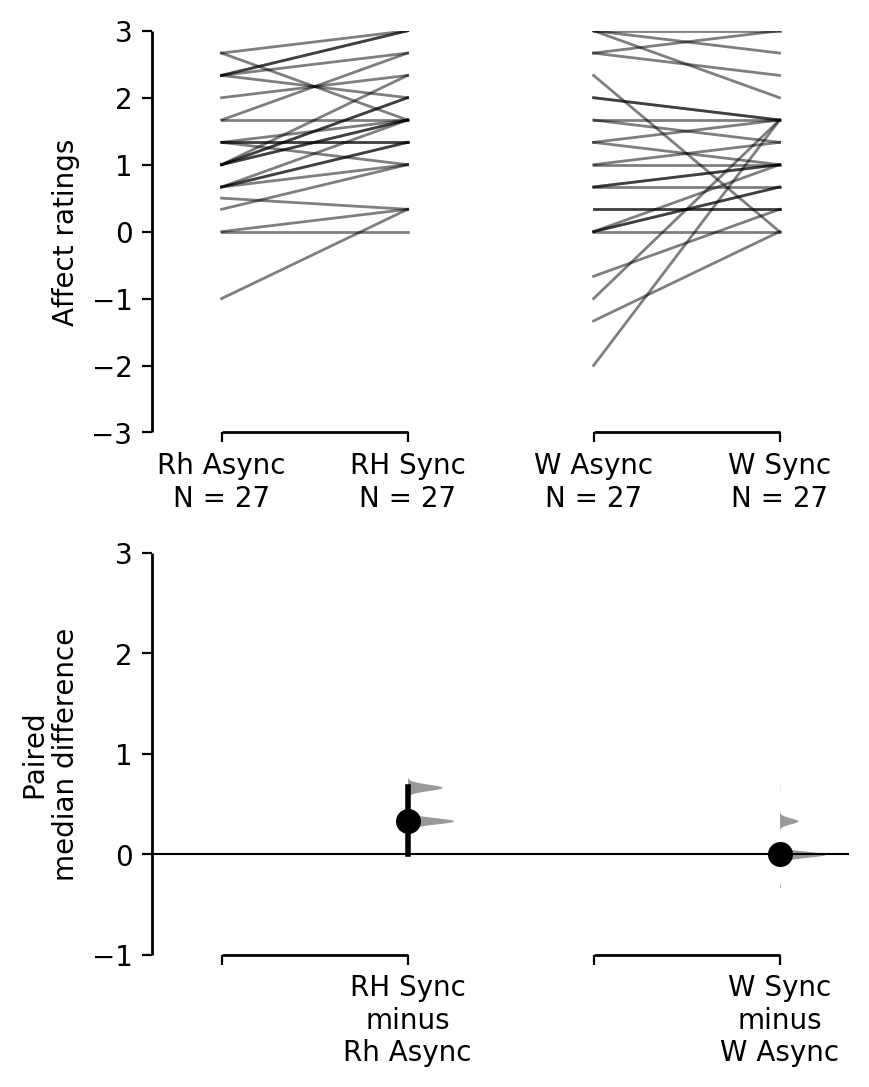


Fig S11 the upper axes show the raw data, that is, the Affect component of the illusion as measured by the rubber hand illusion questionnaire. On the lower axes, the paired median difference between RH Synchronous and RH Asynchronous, and between Wood Synchronous and Wood Asynchronous is plotted as a bootstrap sampling distribution. Median differences are depicted as dots; 95% confidence intervals are indicated by the ends of the vertical error bars.


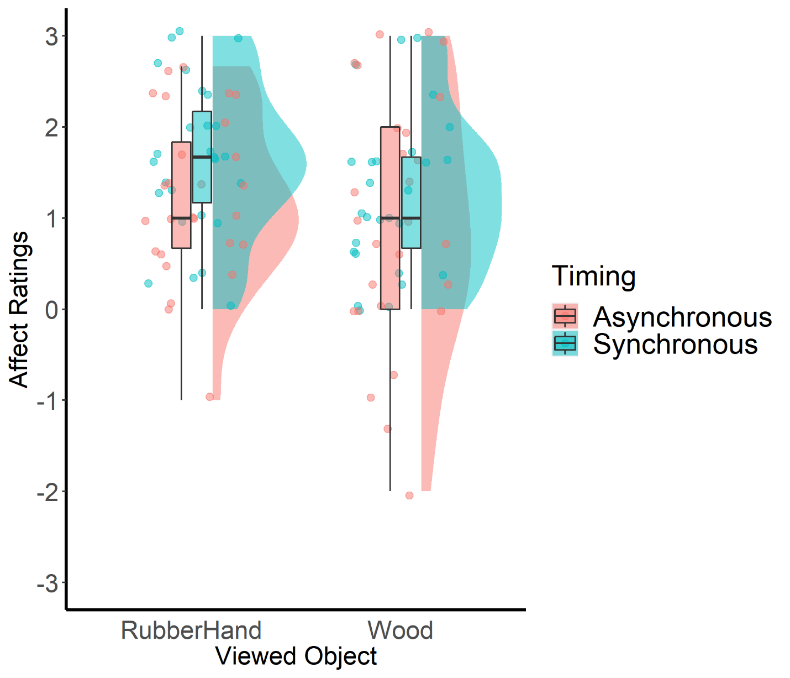


Fig S12 A raincloud plot shows Affect scorings as measured with the rubber hand illusion questionnaire for the four different visuo-tactile stimulations.

### Deafference

Deafference is related to sensations of numbness and pins-and-needles in one’s hand.

With the rubber hand, there was no difference (Me_diff_ = 0; 95% CI [0, 0]) between synchronous (Me = 0.33, IQR = 2.5) and asynchronous (Me = 0, IQR = 1.83) stimulation (see Fig S13).

With the wooden hand, there was no difference (Me_diff_ = 0; 95% CI [0, 0]) between synchronous (Me = -0.66, IQR = 2.16) and asynchronous (Me = -1, IQR = 3.33) stimulation (see Fig S13).

A raincloud plot shows that the Deafference scores did not differ between the rubber hand synchronous and all other experimental conditions (see Fig S14).


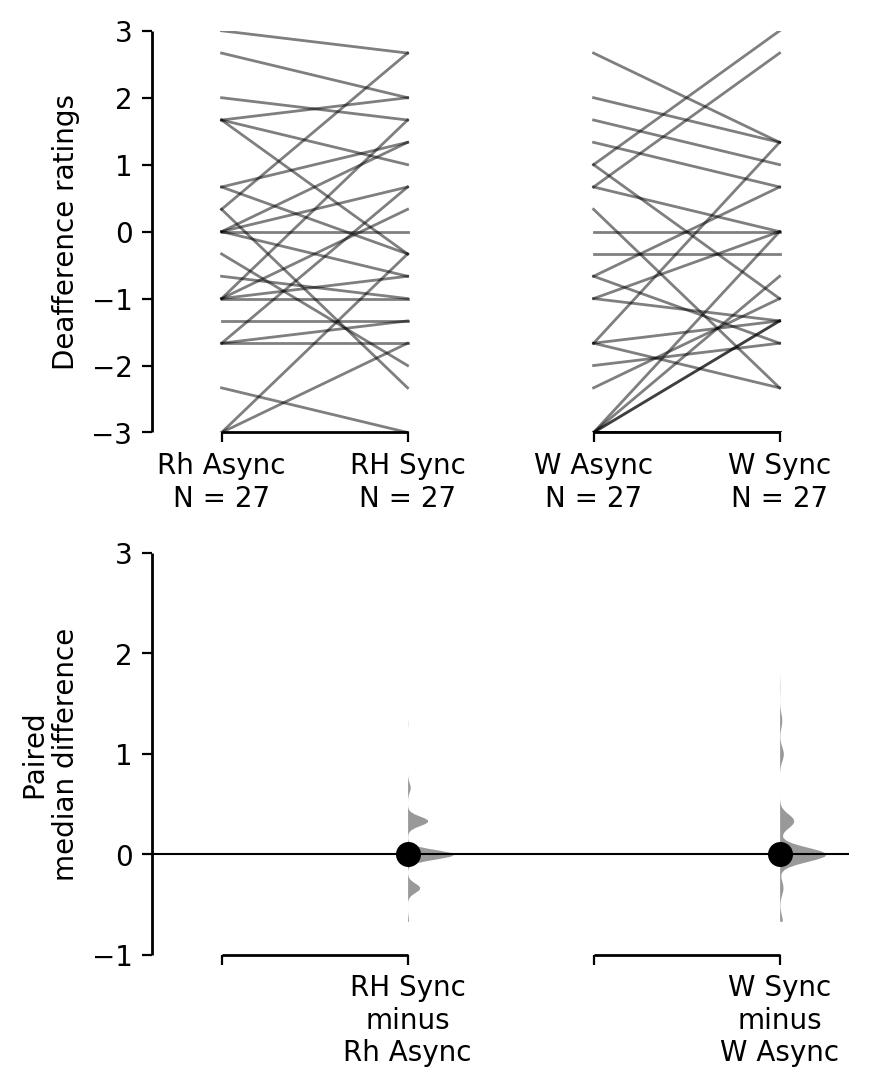


Fig S13 the upper axes show the raw data, that is, the Deafference component of the illusion as measured by the rubber hand illusion questionnaire. On the lower axes, the paired median difference between RH Synchronous and RH Asynchronous, and between Wood Synchronous and Wood Asynchronous is plotted as a bootstrap sampling distribution. Median differences are depicted as dots; 95% confidence intervals are indicated by the ends of the vertical error bars.


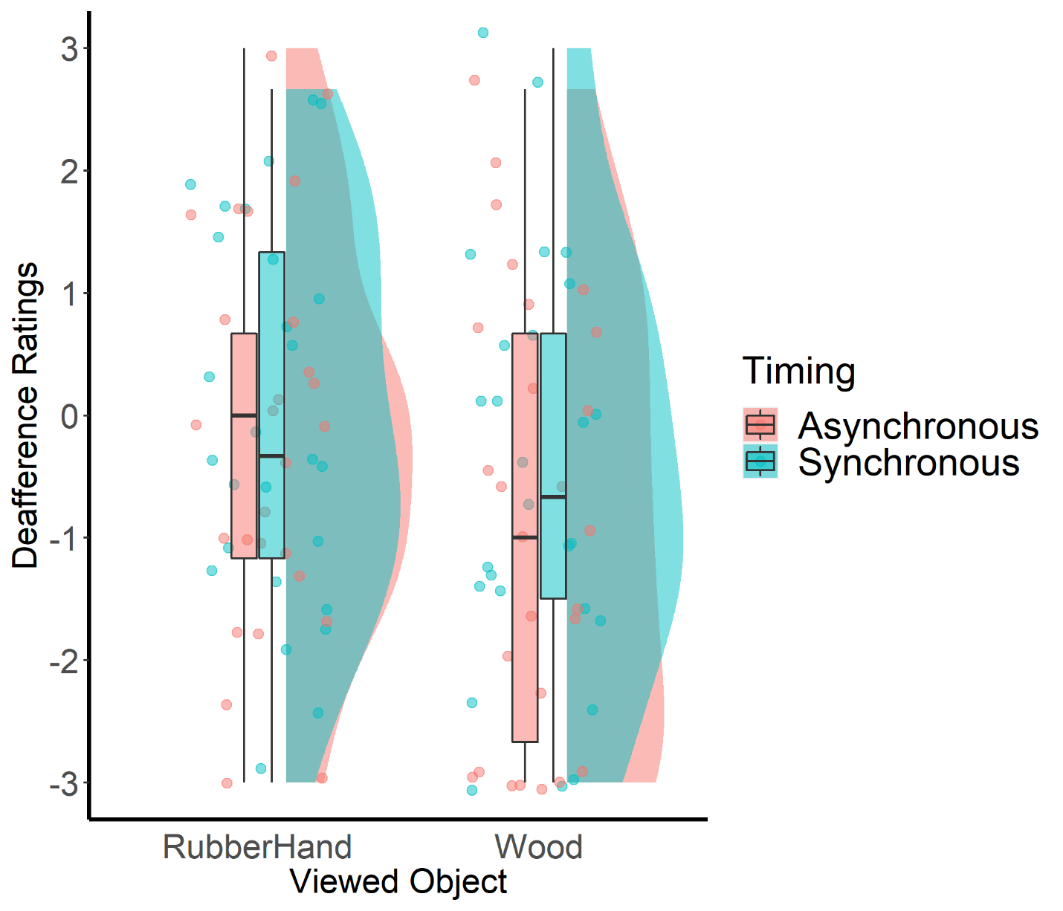


Fig S14 A raincloud plot shows Deafference scorings as measured with the rubber hand illusion questionnaire for the four different visuo-tactile stimulations.

### Location

Location refers to the feeling that the rubber hand and one’s hand are in the same place.

With the rubber hand, there was a moderate difference (Me_diff_ = 1.67; 95% CI [0, 2.33]) between synchronous (Me = 0.33, IQR = 1.83) and asynchronous (Me = -1, IQR = 2.83) stimulation (see Fig S15).

With the wooden hand, there was no difference (Me_diff_ = 0.33; 95% CI [0, 0.66]) between synchronous (Me = -1, IQR = 2.33) and asynchronous (Me = -2.66, IQR = 2.33) stimulation (see Fig S15).

A raincloud plot shows that the Location scores were higher in the rubber hand synchronous than in all other experimental conditions (see Fig S16).


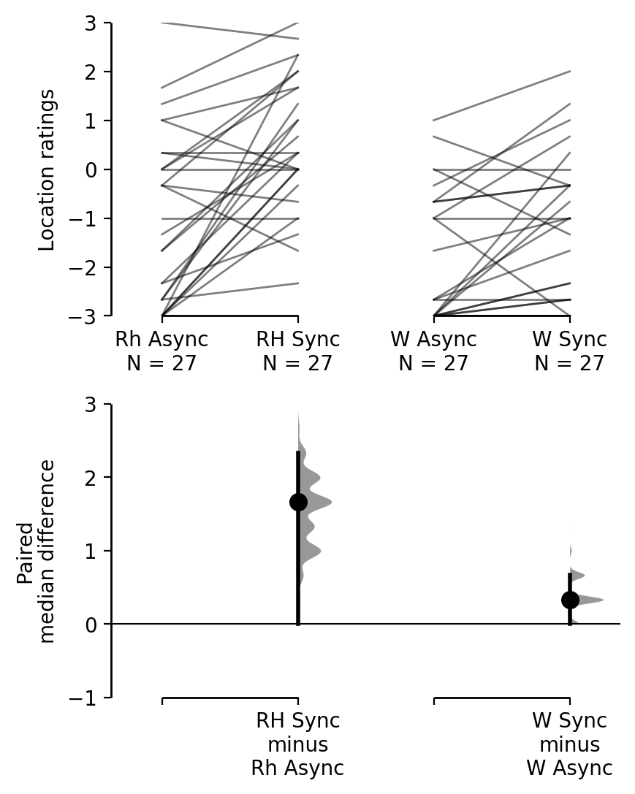


Fig S15 the upper axes show the raw data, that is, the Location component of the illusion as measured by the rubber hand illusion questionnaire. On the lower axes, the paired median difference between RH Synchronous and RH Asynchronous, and between Wood Synchronous and Wood Asynchronous is plotted as a bootstrap sampling distribution. Median differences are depicted as dots; 95% confidence intervals are indicated by the ends of the vertical error bars.


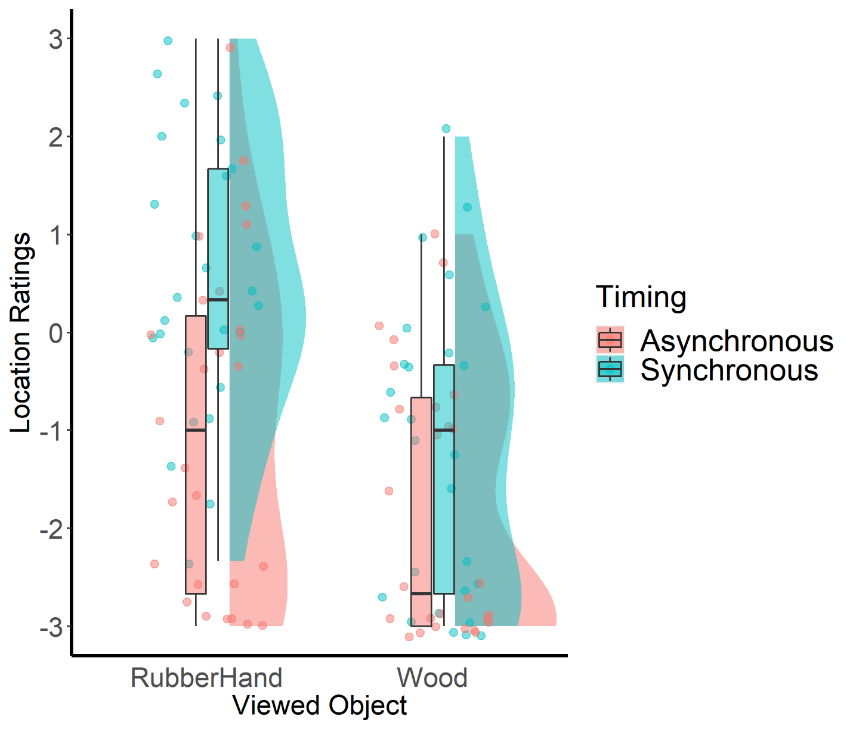


Fig S16 A raincloud plot shows Location scorings as measured with the rubber hand illusion questionnaire for the four different visuo-tactile stimulations.

### Agency

Agency refers to the feeling of being able to move the rubber hand.

With the rubber hand, there was a weak difference (Me_diff_ = 1; 95% CI [0, 1.5]) between synchronous (Me = 0, IQR = 3) and asynchronous (Me = -1, IQR = 2.75) stimulation, indicating a probable effect on this component in this condition (see Fig S17).

With the wooden hand, there was no difference (Me_diff_ = 0; 95% CI [0, 0]) between synchronous (Me = -2, IQR = 2.25) and asynchronous (Me = -2.5, IQR = 3) stimulation, indicating no effect on this component in this condition (see Fig S17).

A raincloud plot shows that the Agency scores were higher in the rubber hand synchronous than in all other experimental conditions (see Fig S18).


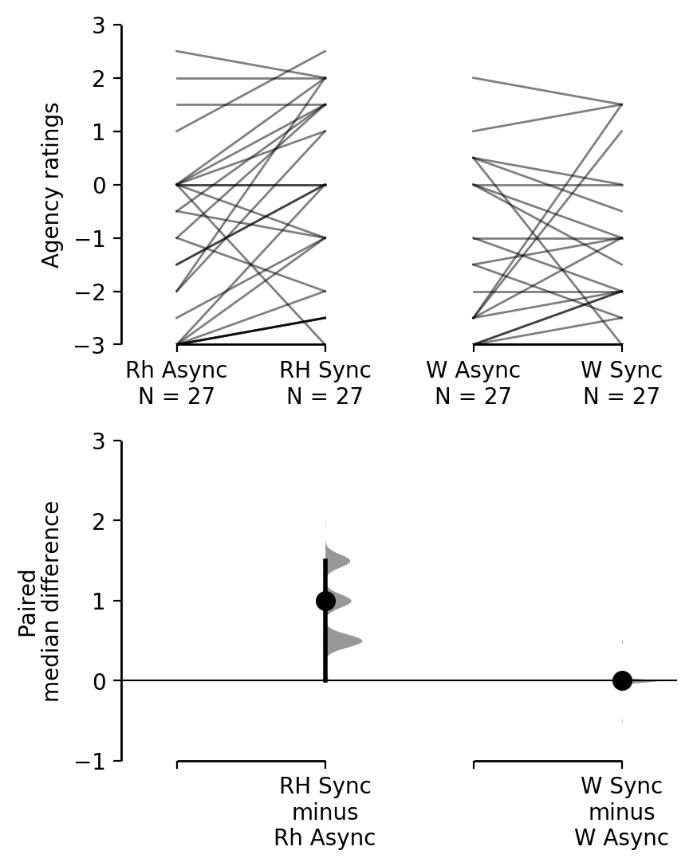


Fig S17 the upper axes show the raw data, that is, the Agency component of the illusion as measured by the rubber hand illusion questionnaire. On the lower axes, the paired median difference between RH Synchronous and RH Asynchronous, and between Wood Synchronous and Wood Asynchronous is plotted as a bootstrap sampling distribution. Median differences are depicted as dots; 95% confidence intervals are indicated by the ends of the vertical error bars.


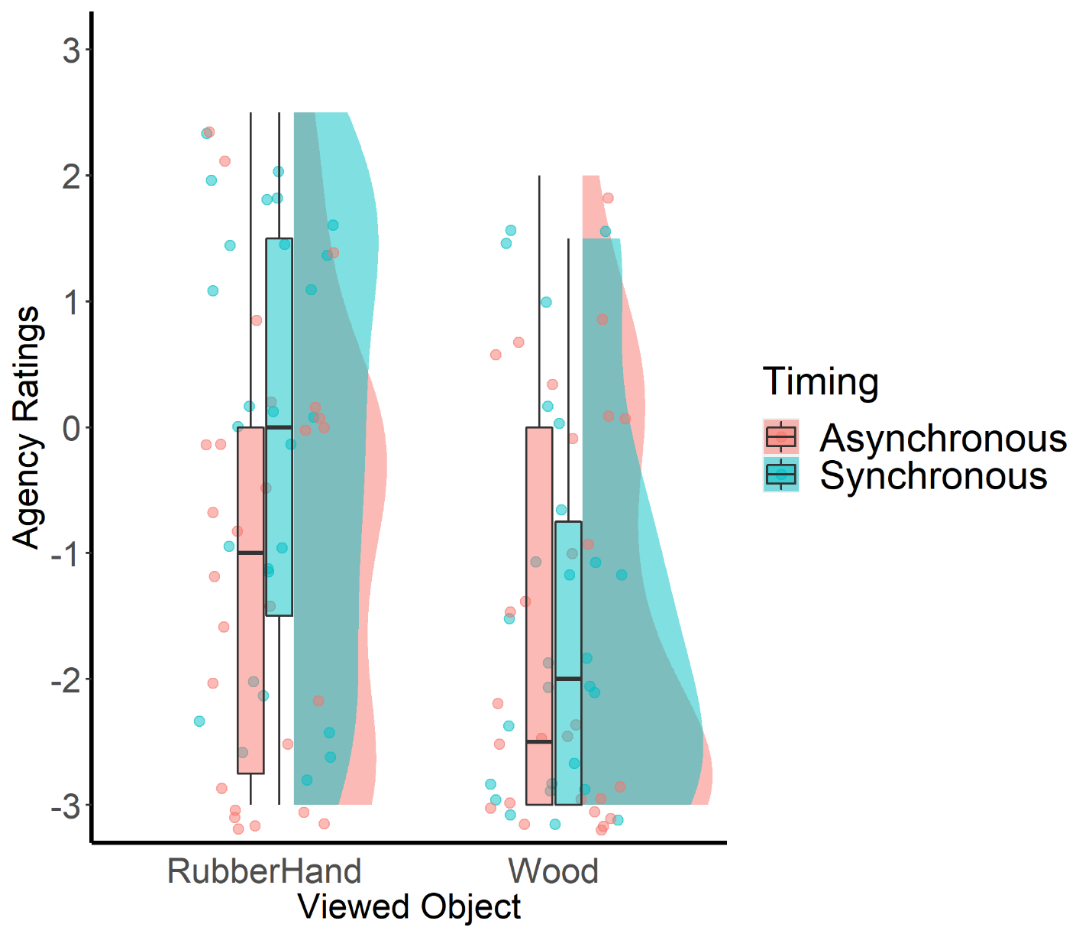


Fig S18 A raincloud plot shows Agency scorings as measured with the rubber hand illusion questionnaire for the four different visuo-tactile stimulations.

## Questionnaires’ results summary

Summarizing, the results of the analysis of the different components of the illusion show that *Embodiment* ratings were higher for the rubber hand synchronous as compared to all other experimental conditions.

Ratings on the Ownership, Location and Agency were higher in the rubber hand synchronous than in all other experimental conditions, however this difference was weak.

There was no difference between conditions in all the other components. These results suggest that, at a group level, the synchronous visuo-tactile stimulation was successful in eliciting the explicit component of the rubber hand illusion as participants felt that the rubber hand belonged to their body (*Embodiment*) and that their real stimulated hand was closer to the rubber hand.

## Rubber Hand Illusion Questionnnaire Items

Table 1 Rubber Hand Illusion questionnaire. The second column shows which component and subcomponent each items belonged to.

|  | Component | Sub-components |
| --- | --- | --- |
| It seemed like I was looking directly at my  own hand, rather than at a rubber hand. | Embodiment | Ownership |
|  |  |  |
| It seemed like the rubber hand began to resemble my real hand. | Embodiment | Ownership |
|  |  |  |
| It seemed like the rubber hand belonged to me. | Embodiment | Ownership |
|  |  |  |
| It seemed like the rubber hand was my hand. | Embodiment | Ownership |
|  |  |  |
| It seemed like the rubber hand was part of my body. | Embodiment | Ownership |
|  |  |  |
| It seemed like my hand was in the location where the rubber hand was. | Embodiment | Location |
|  |  |  |
| it seemed like the rubber hand was in the location where my hand was. | Embodiment | Location |
|  |  |  |
| It seemed like the touch I felt was caused by the paintbrush touching the rubber hand. | Embodiment | Location |
|  |  |  |
| It seemed like I could have moved the rubber hand if I had wanted. | Embodiment | Agency |
|  |  |  |
| It seemed like I was in control of the rubber hand. | Embodiment | Agency |
|  |  |  |
| II seemed like I was unable to move my hand. | Loss of own hand |  |
|  |  |  |
| II seemed like I could have moved my hand if I had wanted. | Loss of own hand |  |
|  |  |  |
| It seemed like I couldn’t really tell where my hand was. | Loss of own hand |  |
|  |  |  |
| It seemed like my hand had disappeared. | Loss of own hand |  |
|  |  |  |
| It seemed like my hand was out of my control. | Loss of own hand |  |
|  |  |  |
| It seemed like my hand was moving towards the rubber hand. | Movement |  |
|  |  |  |
| It seemed like the rubber hand was moving towards my hand. | Movement |  |
|  |  |  |
| It seemed like I had three hands. | Movement |  |
|  |  |  |
| I found that experience enjoyable. | Affect |  |
|  |  |  |
| I found that experience interesting. | Affect |  |
|  |  |  |
| The touch of the paintbrush on my finger was pleasant. | Affect |  |
|  |  |  |
| I had the sensation of pins and needles in my hand. | Deafference |  |
|  |  |  |
| I had the sensation that my hand was numb. | Deafference |  |
|  |  |  |
| It seemed like the experience of my hands was less vivid than normal. | Deafference |  |

# Tests of normality

Before running the statistical analyses, we checked whether our variables of interest were normally distributed by visual inspection of density distributions to check the Skewness (that is, the symmetry of the data) and Kurtosis (that is, whether the data are heavy-tailed, or light-tailed).

The Ownership Potentiometer Ratings were highly Skewed whereas the Proprioceptive Drift were normally distributed; therefore, we used respectively the Median and the Mean to analyze these variables.

All the scales of the RHI questionnaire were not normally distributed, except for the Deafference scale. For consistency, we used the Median in the analyses for all the questionnaire scales and subscales.

Following, we include density plots for our main variables in this order: Ownership Potentiometer Ratings, Proprioceptive Drifts, Embodiment, Ownership, Loss, Movement, Affect, Deafference, Location, and Agency.


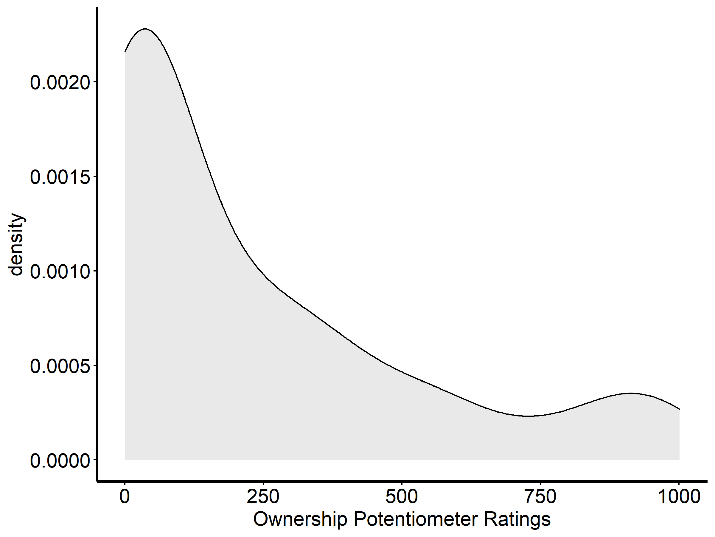

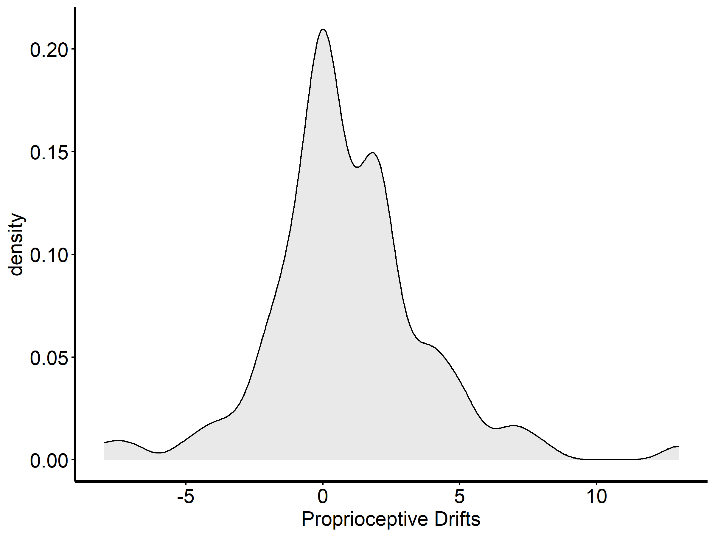


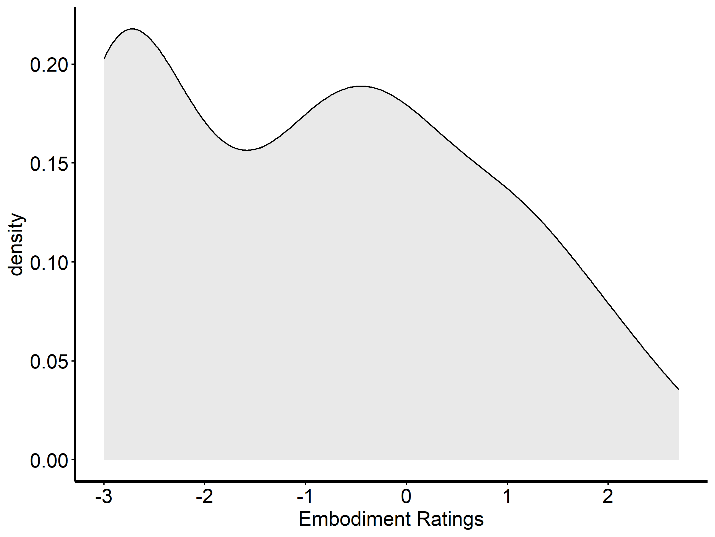

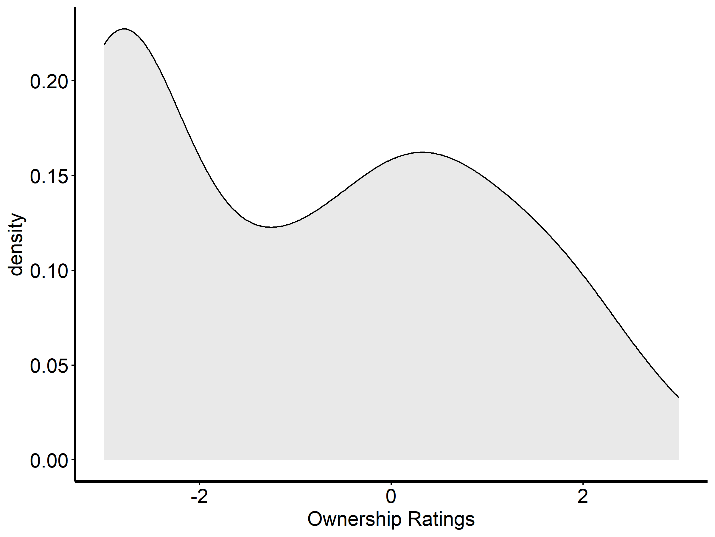


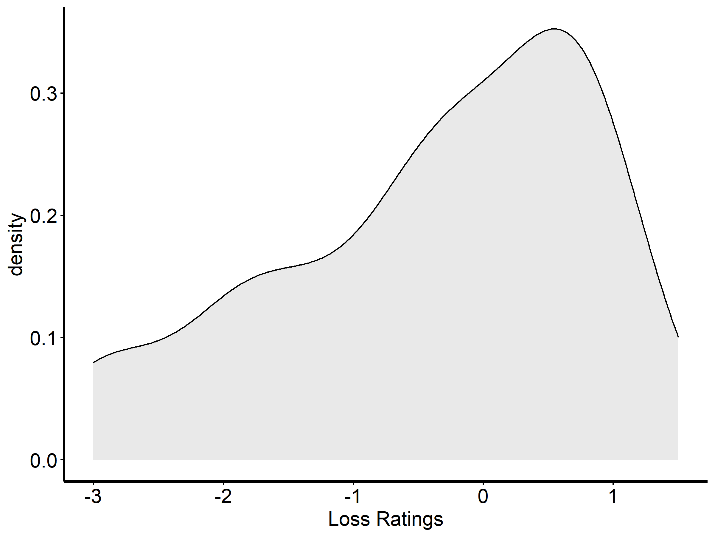

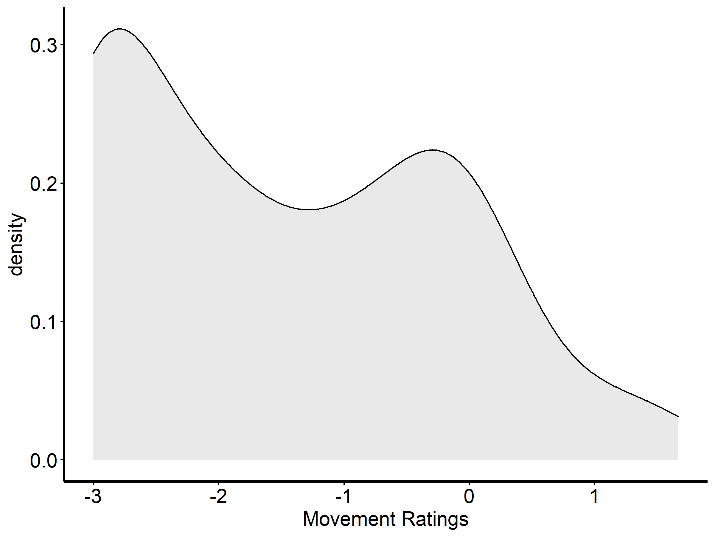

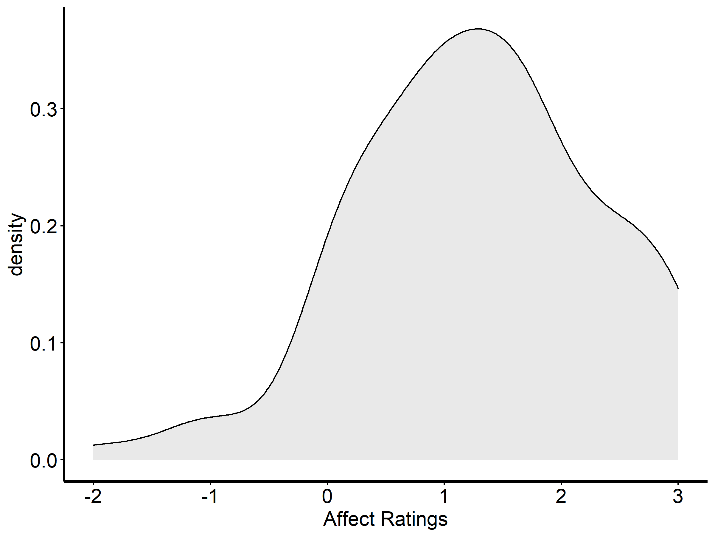

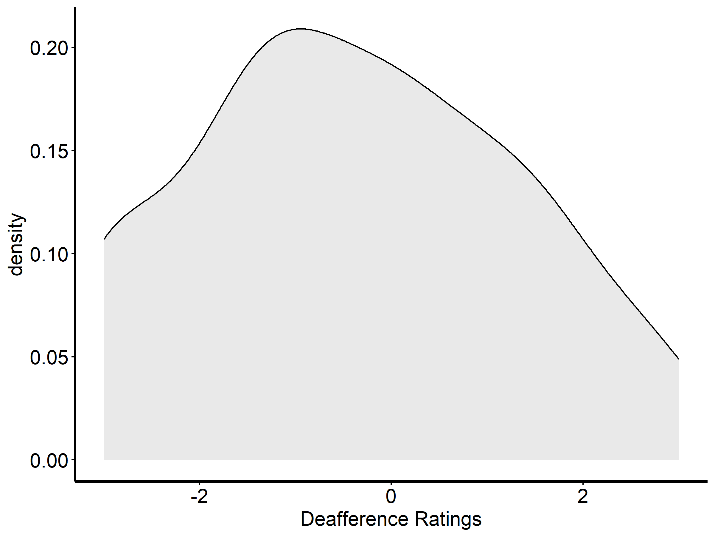

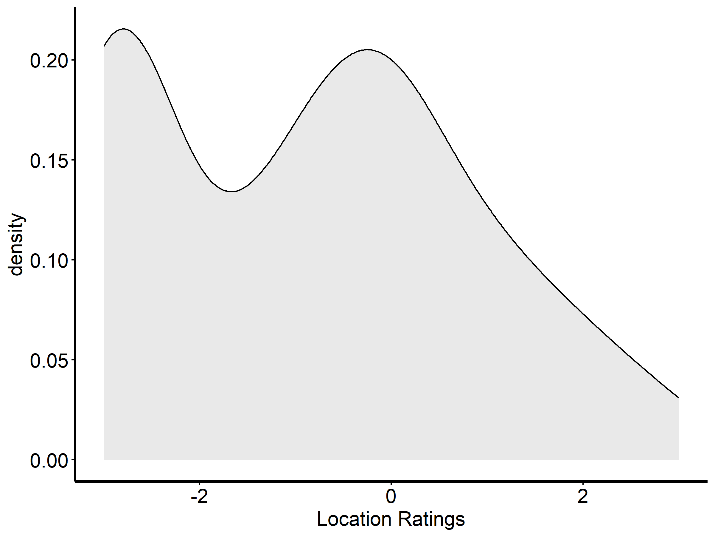

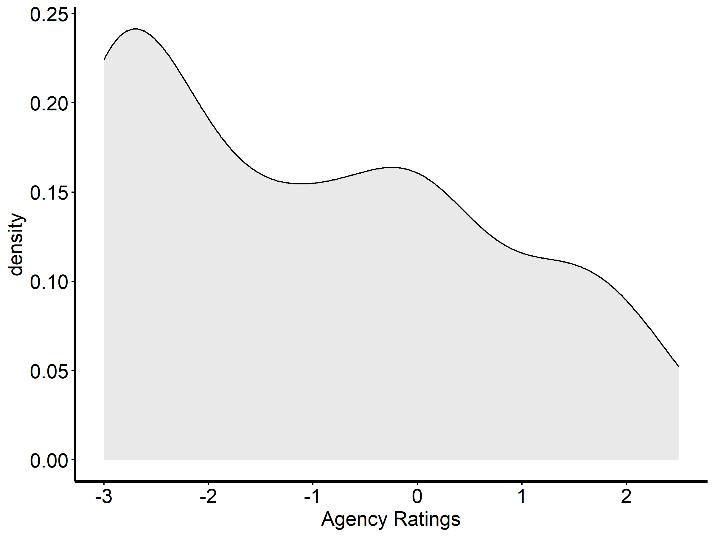


# Bibliography

1. Tsakiris, M. My body in the brain: a neurocognitive model of body-ownership. *Neuropsychologia* 48, 703–12 (2010).

2. Longo, M. R., Schüür, F., Kammers, M. P. M., Tsakiris, M. & Haggard, P. What is embodiment? A psychometric approach. *Cognition* 107, 978–998 (2008).
